# Profiles of Fatigue and Psychological Symptoms in Long‐Term Childhood, Adolescent, and Young Adult Cancer Survivors—The NOR‐CAYACS Study

**DOI:** 10.1002/cam4.70425

**Published:** 2024-11-25

**Authors:** Wei H. Deng, Hanne C. Lie, Ellen Ruud, Jon H. Loge, Cecilie E. Kiserud, Corina S. Rueegg

**Affiliations:** ^1^ Oslo Centre for Biostatistics and Epidemiology Oslo University Hospital Oslo Norway; ^2^ Oslo Centre for Biostatistics and Epidemiology, Department of Biostatistics, Institute of Basic Medical Sciences University of Oslo Oslo Norway; ^3^ National Advisory Unit on Late Effects After Cancer Treatment Oslo University Hospital Oslo Norway; ^4^ Department of Behavioral Medicine, Institute of Basic Medical Sciences University of Oslo Oslo Norway; ^5^ Department of Paediatric Haematology and Oncology, Division for Paediatric and Adolescent Medicine Oslo University Hospital Oslo Norway; ^6^ Faculty of Medicine, Institute for Clinical Medicine University of Oslo Oslo Norway; ^7^ Epidemiology, Biostatistics and Prevention Institute University of Zurich Zurich Switzerland

**Keywords:** CAYACS, fatigue, HRQOL, latent profile analysis, long‐term survivors, psychological symptoms

## Abstract

**Introduction:**

Long‐term childhood, adolescent, and young adult cancer survivors (CAYACS) are at risk of fatigue and psychological problems. However, their interactions remain largely unexplored. Understanding how they cluster can inform treatment and person‐centered follow‐up care. We aimed to identify and describe profiles of co‐occurring fatigue and psychological symptoms and investigate their associations with health‐related quality of life (HRQOL) in CAYACS.

**Methods:**

NOR‐CAYACS (The Norwegian Childhood, Adolescents and Young Adult Cancer Survivors study) was a nationwide survey involving adult survivors of any childhood cancer (aged < 19 years at diagnosis) and selected young adult cancers (breast and colorectal cancers, non‐Hodgkin lymphoma, leukemias, and malignant melanomas, aged 19–39 years at diagnosis) identified through the Cancer Registry of Norway. We included 1893 survivors aged ≥ 18 years, ≥ 5 years since diagnosis. We performed latent profile analysis with six continuous outcomes: physical and mental fatigue, depression, anxiety, post‐traumatic stress symptoms, and fear of recurrence.

**Results:**

We identified an overall “Low” (64%), a “Moderate fatigue/high anxiety” (18%), a “High fatigue/moderate distress” (13%), and an overall “High” (5%) symptom burden profile. The “High” profile exhibited lowest physical‐ and mental‐HRQOL with T‐scores −9.8 (95% confidence interval [95% CI]: −12.5, −7.1) and −25.0 (95% CI: −26.7, −23.3) compared to the “Low” profile.

**Conclusion:**

We identified four profiles, two characterized by high fatigue and two near normative fatigue levels, each with different psychological symptom burden. Greater symptom burden corresponded to lower HRQOL, with high fatigue profiles showing lower physical HRQOL. These profiles help identify at‐risk individuals and allow for targeting interventions and follow‐up care to survivors' unique constellation of symptoms.

## Graphical abstract

Our study identified four unique profiles of fatigue and psychological symptoms in childhood, adolescent, and young adult cancer survivors. Profiles of symptom burden provide insight for the development of multimodal interventions targeting cancer survivors' unique constellation of cancer burden.

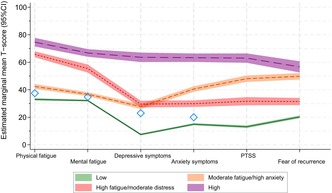

## 1 Introduction

Long‐term childhood, adolescent, and young adult cancer survivors (CAYACS) are at risk of fatigue, often accompanied by multiple psychological symptoms [1, 2]. With survival rates over 90% for childhood cancers and 80% for young adult cancers, understanding late effects in this population becomes increasingly important [3, 4]. While prevalent, the full spectrum of fatigue's associations with psychological symptoms remains largely unexplored. Fatigue has been associated with depressed mood, anxiety, cognitive impairment, and emotional distress, but primarily as single correlations and not as profiles of co‐occurring symptoms [5, 6]. These symptoms moreover likely exacerbate each other [7], with potentially higher negative impact on health‐related quality of life (HRQOL) when present simultaneously [8]. However, a comprehensive understanding of how fatigue and other psychological symptoms cluster at the individual survivor's level is currently lacking [1, 9, 10, 11].

The European Commission's Joint Action on Cancer Control's (CanCon) recommendations for rehabilitation and survivorship advise a person‐centered approach using empirical data on survivors' risk profiles to organize care and tailor health plans [12]. Yet, less than half of adult long‐term childhood cancer survivors in most high‐income countries receive follow‐up care [13], with even fewer receiving care for psychological issues [12]. Insights into how fatigue and psychological symptoms cluster can lead to more effective treatment strategies and person‐centered, risk‐stratified follow‐up care for the diverse CAYACS population.

Addressing this landscape of symptoms and their relationships requires robust profiling methods. Latent profile analysis (LPA) is a valuable tool to characterize CAYACS into distinct unobserved (latent) groups, so‐called profiles (distinct homogenous subgroups within a heterogenous population) [14]. Unlike traditional cluster analysis, it offers a more statistically robust model‐based approach for identifying profiles across multiple continuous outcomes, without relying on arbitrary cutoffs, composite scores, or subjective cluster numbers [15]. Previous researches that have used LPA for psychological outcomes in CAYACS identified two to four profiles [9, 16, 17, 18, 19, 20, 21], where larger studies more often found four [9, 16, 17, 20]. All but two involved survivors of childhood cancer [9, 16, 17, 18, 19], with the remaining two in breast cancer survivors [20, 21]. Five of these studies were conducted in North America [9, 16, 17, 18, 20] and two in East Asia [19, 21]. However, none investigated fatigue and psychological symptoms in European countries nor across the whole age range of CAYACS or multiple diagnoses among survivors of young adult cancers.

Our aim was therefore to (1) identify profiles of fatigue and psychological symptoms in a large nationwide sample of adult long‐term CAYACS through LPA; (2) describe sociodemographic and cancer‐related background characteristics of identified profiles; and (3) investigate the association between identified profiles and HRQOL.

## 2 Methods

This paper is written in accordance with the Strengthening the Reporting of Observational Studies in Epidemiology (STROBE) guidelines [22].

### 2.1 Study Design and Participants

The Norwegian Childhood, Adolescents and Young Adult Cancer Survivors study (NOR‐CAYACS) was a cross‐sectional, population‐based questionnaire study, in which eligible 5‐year survivors were identified via the Cancer Registry of Norway (CRN, *n* = 5361) [23]. Inclusion criteria were: ≥ 18 years of age at study, diagnosed between 1985 and 2009 of either any childhood cancer (diagnosed before age 19, except central nervous system tumors due to uncertainty of current cognitive functioning), or specific young adult (diagnosed ages 19–39) cancers: breast (ICD‐10: C50, stages≤III), colorectal (ICD‐10: C18–20), non‐Hodgkin lymphoma (NHL, ICD‐10: C82–85), leukemia (ICD‐10: C91–96), or a random sample of malignant melanoma (ICD‐10:C43). We excluded Hodgkin's lymphoma, testicular, and cervical cancer in young adults, as these were already enrolled in other concurrent studies at our center.

Eligible participants were mailed the questionnaire and an informed consent form between 2015 and 2016, of whom 2104 responded. We excluded 86 participants diagnosed with a new cancer within the last 5 years (after 2009) according to the CRN, to meet the inclusion criteria of being a 5‐year survivor, and 125 who self‐reported undergoing active cancer treatment, leaving 1893 participants for analyses (Figure S1).

The study was approved by The Norwegian Data Protection Authority (15/00395‐2/CGN), the Regional Committee for Medical Research Ethics (2015/232 REK sør‐øst B), and the Data Protection Officer at Oslo University Hospital.

### 2.2 Measures of Fatigue and Psychological Symptoms

We used six continuous outcomes [physical and mental fatigue, depressive symptoms, anxiety symptoms, post‐traumatic stress symptoms (PTSS), and fear of cancer recurrence] from five validated self‐reported questionnaires, with all scales standardized to T‐scores from 0 to 100 for comparability. All outcomes were scored following their respective manuals, which included handling of missing data if provided [24, 25, 26, 27, 28]. Only the Hospital Anxiety and Depression Scale (HADS) manual included instructions for imputing single missing items [29]. For reference, we present Norwegian normative scores for fatigue, depressive symptoms, and anxiety symptoms [30, 31]. There are no normative values for PTSS and fear of recurrence. More details are in the Supplementary Methods (Supplement, Chapter A).

We used Chalder's Fatigue Questionnaire to assess fatigue severity the past month [24, 32, 33]. It consists of 11 items summed into a *physical* (Cronbach's alpha for included survivors: *α* = 0.90) and *mental fatigue* (*α* = 0.82) subscale. Items were scored 0–3 where higher scores represent higher severity. Subscales were set to missing if any item was missing (3.3% of participants).

Severity of *depressive symptoms* the past 2 weeks was assessed using the nine‐item Patient Health Questionnaire (PHQ‐9; *α* = 0.84) [25]. Each item ranges from 0 (not at all) to 3 (nearly every day) and corresponds to the nine diagnostic criteria for major depressive disorder in the Diagnostic and Statistical Manual of Mental Disorders V. We used a modified symptom severity score from the sum of emotional and cognitive symptom items to avoid potential overlap with non‐depression‐related fatigue (anhedonia, depressed mood, feelings of worthlessness, poor concentration, and thoughts of self‐harm/suicidal ideations) [34]. The PHQ‐9 depressive symptoms score allows for mean substitution if only one item is missing. A missing score was assigned 0.4% of participants due to missing all five items; all other participants had complete scores [25].

*Anxiety symptoms* severity (*α* = 0.83) was measured using the anxiety subscale of The HADS questionnaire [26, 29]. It comprises the sum of seven items ranging from 0 (not present) to 3 (highly present) and covers symptoms experienced the previous week. Individual subscale means were imputed if less than half the scale items were missing or otherwise set to missing (0.3%) [35].

Cancer‐related *PTSS* (*α* = 0.87) was measured using the Impact of Event Scale‐6 (IES‐6), which maps intrusive thoughts and avoidance behavior the past week [28, 36]. It is scored as the sum of six items, each ranging from 0 (not at all) to 4 (extremely) according to symptom intensity. Participants with any missing items were set as missing (1.9%).

Fear of cancer recurrence (further called *fear of recurrence*) was measured with the three relevant items of the cancer worry subscale of the Assessment of Survivor Concerns (ASC) questionnaire (*α* = 0.84) [27]. These items assess survivors' worry about future medical tests, cancer recurrence, and new cancers on a 4‐point Likert scale from 1 (not at all) to 4 (a lot). Participants missing any item were set as missing (0.7%).

### 2.3 Health‐Related Quality of Life

We used the Short‐Form 12 (SF‐12) to assess self‐reported HRQOL in two summary scores: *physical* and *mental HRQOL* [37]. Raw scores were converted into T‐scores (mean = 50, standard deviation (SD) = 10) according to the US standard population provided in the manual, where higher scores indicate better HRQOL. We set 4% as missing due to at least one missing item.

### 2.4 Covariates

The CRN provided clinical and demographic data including sex, birthdate, date of first diagnosis, primary diagnosis (classified as childhood cancer, melanoma, breast cancer, colorectal cancer, non‐Hodgkin's lymphoma, and leukemia), and second cancer(s). Childhood or adolescent cancers (< 19 years) were further stratified into leukemias, lymphomas, bone and soft tissue sarcomas, germ cell tumors, and other tumors. We calculated age at study and time since first cancer diagnosis through to 15th May 2015. Cancer treatment was self‐reported and hierarchically classified into minimal treatment for melanoma (local treatment only and diagnosis of melanoma), local treatment (local radiotherapy or surgery only), systemic single treatment (including chemotherapy, stem cell transplantation, and immune therapy), and multiple treatments [38]. Highest achieved education (compulsory school up to 9 years, vocational school, high school, and university), and recurring cancer(s) (relapse or second cancers; dichotomized along with second cancers from the CRN as none or ≥ 1) were also self‐reported [39].

### 2.5 Statistical Analysis

Descriptive statistics were presented as means with SDs for continuous variables and frequencies with proportions for categorical variables).

We used LPA to identify symptom profiles across the six continuous outcome measures [15, 40]. LPA is a type of a finite mixture model that assumes that the population heterogeneity for these outcomes stems from homogenous subgroups (latent profiles) with similar experiences [14]. We ran models from two to seven profiles (subgroups), fit using maximum likelihood estimation. Every individual's probability of belonging to each profile was thereafter calculated (posterior probability). The models used all available information, and only participants with all outcomes missing were excluded (none in our analyses).

The best model regarding number of profiles was quantitatively evaluated with fit indices: primarily Bayesian information criterion (BIC) [40], entropy (reflecting how distinct profiles are from each other, ranging from 0‐little to 1‐high), lowest estimated proportion of participants in a profile being ≥ 5%, and average maximal posterior probability [41]. We additionally qualitatively evaluated profiles based on clinical interpretability. A Sankey diagram is provided to illustrate how survivors were reallocated with an increasing number of profiles. The Supplementary Methods contain further details on the fit indices (Supplement, Chapter A).

We described sociodemographic and cancer‐related background characteristics for the final profiles identified, where participants were assigned the profile with the highest posterior probability (modal assignment). *p*‐values for comparisons across profiles were calculated using univariable linear regression models for continuous characteristics and chi‐squared statistics for categorical characteristics.

We conducted univariable and multivariable linear regression models to investigate the association between profile membership and physical and mental HRQOL. Covariates were selected based on a Directed Acyclic Graph (DAG) and included age at study, sex, highest achieved education, primary diagnosis, cancer treatment, time since first diagnosis, and recurring or second cancer(s) (Figure S2). To account for the uncertainty of profile allocation, each participant was assigned all four profiles with weights based on their posterior probabilities of profile membership, calculating robust 95% confidence intervals (95% CI) clustered on each individual. Participants with missing cancer treatment information (*n* = 97) were kept in the model and coded as a separate category. Participants with missing HRQOL (*n* = 69) or any covariate data (education, *n* = 10) were excluded (*n* = 79) from all models.

For sensitivity analyses, we ran the LPA and description of related background characteristics for the following three subgroups: (1) sample excluding melanoma survivors (*n* = 1595), (2) childhood cancer survivors (CCS) only (including child and adolescent cancer patients diagnosed < 19 years, *n* = 598), and (3) young adult cancer survivors (YACS) only (including patients diagnosed 19–39 years, *n* = 1295).

All analyses were conducted in STATA v18 with a *p*‐value ≤ 0.05 considered statistically significant.

## 3 Results

### 3.1 Participant Characteristics

Mean age at study was 30.2 years (SD 7.9) for the 598 CCS and 49.0 years (SD 7.8) for the 1295 YACS (Table 1). The proportion of female participants was higher in both CCS (56%) and YACS (73%). Most CCS were leukemia survivors (32%), and breast cancer was the most frequent diagnosis among YACS (38%). The average time since first diagnosis was 19.1 (SD 6.6) years in CCS and 15.6 (SD 6.7) years in YACS. Most (58%) received multiple treatments, 22% of YACS received minimal treatment for melanoma, and overall 3% experienced recurring or second cancer(s).

**TABLE 1:** Background characteristics by age group at diagnosis and primary diagnosis (*N* = 1893).

	Age group at diagnosis		Diagnosis (young adult cancer survivors)					
	Childhood cancer	Young adult cancer	Melanoma	Breast cancer	Colorectal	NHL	Leukemia	Total
	*N* = 598	*N* = 1295	*N* = 298	*N* = 488	*N* = 143	*N* = 230	*N* = 136	*N* = 1893
**Background**								
Age at study (yrs)	30.2 (±7.9)	49.0 (±7.8)	49.5 (±8.1)	49.7 (±6.9)	48.7 (±9.0)	48.6 (±8.2)	46.6 (±8.2)	43.1 (±11.7)
Sex, female	337 (56%)	943 (73%)	213 (71%)	488 (100%)	73 (51%)	108 (47%)	61 (45%)	1280 (68%)
Highest achieved education								
Compulsory school	31 (5%)	80 (6%)	13 (4%)	27 (6%)	9 (6%)	15 (7%)	16 (12%)	111 (6%)
Vocational school	134 (22%)	324 (25%)	69 (23%)	127 (26%)	31 (22%)	62 (27%)	35 (26%)	458 (24%)
High school	102 (17%)	122 (9%)	31 (10%)	47 (10%)	14 (10%)	19 (8%)	11 (8%)	224 (12%)
University	329 (55%)	761 (59%)	183 (62%)	283 (58%)	89 (62%)	133 (58%)	73 (54%)	1090 (58%)
**Cancer‐related**								
Age at diagnosis (yrs)	10.5 (±6.0)	32.8 (±5.3)	31.4 (±5.7)	35.2 (±3.5)	33.7 (±4.9)	31.0 (±5.6)	29.4 (±5.9)	25.8 (±11.8)
Time since first diagnosis (yrs)	19.1 (±6.6)	15.6 (±6.7)	17.5 (±6.9)	13.9 (±5.9)	14.4 (±7.4)	17.0 (±6.9)	16.7 (±6.3)	16.7 (±6.9)
Primary diagnosis								
Leukemias	191 (32%)							191 (10%)
Lymphomas	144 (24%)							144 (8%)
Bone and soft tissue sarcomas	74 (12%)							74 (4%)
Germ cell tumors	67 (11%)							67 (4%)
Other	122 (20%)							122 (6%)
Melanoma		298 (23%)						298 (16%)
Breast cancer		488 (38%)						488 (26%)
Colorectal cancer		143 (11%)						143 (8%)
Non‐Hodgkin's lymphoma		230 (18%)						230 (12%)
Leukemia		136 (11%)						136 (7%)
Treatment^a^								
Minimal treatment (melanoma)	0 (0%)	262 (22%)	262 (93%)	0 (0%)	0 (0%)	0 (0%)	0 (0%)	262 (15%)
Local treatment	69 (12%)	127 (11%)	0 (0%)	36 (8%)	81 (59%)	10 (5%)	0 (0%)	196 (11%)
Systemic single treatment	162 (28%)	132 (11%)	9 (3%)	3 (1%)	2 (1%)	44 (20%)	74 (59%)	294 (16%)
Multiple treatments	356 (61%)	688 (57%)	11 (4%)	409 (91%)	55 (40%)	161 (75%)	52 (41%)	1044 (58%)
Recurring or second cancer(s)	8 (1%)	54 (4%)	9 (3%)	27 (6%)	1 (1%)	14 (6%)	3 (2%)	62 (3%)

*Note:* Data are presented as mean (±SD) for continuous variables and *n* (%) for categorical variables. Childhood cancers are diagnosed < 19 years, and young adult cancers are diagnosed 19 to 39 years. Missings: Education level [10], Treatment [97].

Abbreviations: *N*, number; NHL, non‐Hodgkin's lymphoma; SD, standard deviation; yrs, years.

^a^ Treatment was hierarchically aggregated into minimal treatment for melanoma (local treatment only and diagnosis of melanoma), local treatment (local radiotherapy or surgery only), systemic single treatment (including chemotherapy, stem cell transplantation, and immune therapy), or multiple treatments.

### 3.2 LPA

T‐ and raw scores of fatigue and psychological symptoms are presented in Table S1 by age group at diagnosis, primary diagnosis, and overall. In the LPA, 2% of participants were missing one outcome, 3% two outcomes, 0.3% three outcomes, 0.2% four outcomes, and one participant was missing five outcomes. The four‐profile model was considered optimal based on clinical interpretability and statistical fit (Table S2), with average posterior probabilities between 0.86 and 0.96 (Table S3).

**FIGURE 1:**
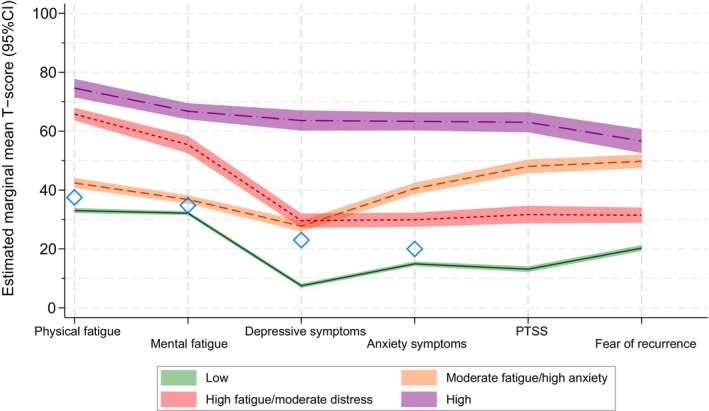
Estimated marginal mean T‐scores for fatigue and psychological symptom burdens for the four identified latent profiles (*N* = 1893). Figure 1 shows the marginal mean T‐scores (darker solid and dashed lines) with 95% confidence intervals (light colored areas) for each of the four identified latent fatigue and psychological symptom profiles. T‐score is a standardized scale from 0 to 100 for all outcomes. Exact estimates are provided in Table 2. **◇** Normative scores for fatigue, depressive symptoms, and anxiety symptoms from Norwegian populations (Dahl et al.; Grov et al.) [30, 31]. PTSS, post‐traumatic stress symptoms.

**FIGURE 2:**
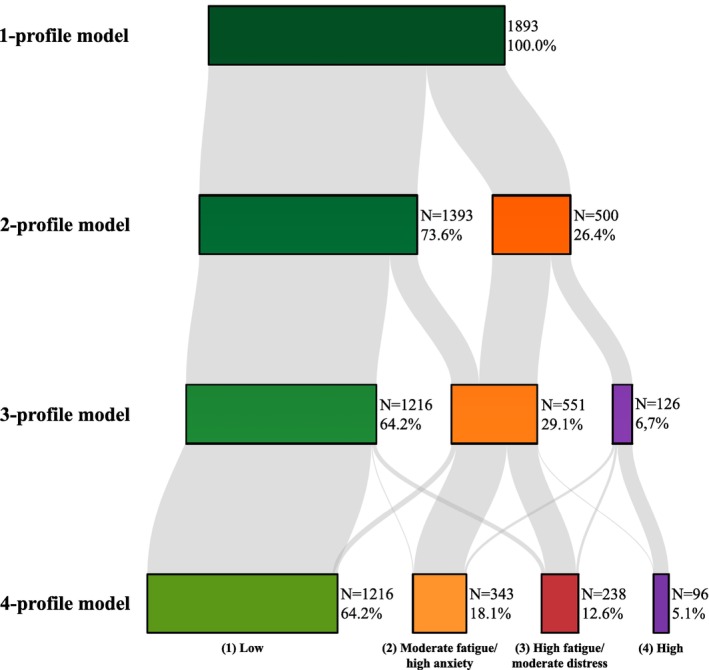
Sankey diagram of reallocation of survivors with the increasing number of latent profiles. Figure 2 shows reallocation of sample individuals by assignment to profile with highest probability (modal assignment) with the increasing number of profiles identified. Each latent profile analysis was run until the model converged. Percentages represent the proportion of each profile within each model. *N*, number of participants in each profile.

The LPA characterized 1216 (64.2%) survivors as overall “low symptom burden (*Low*)”, 343 (18.1%) as “moderate fatigue, high anxiety‐related symptoms (*Moderate fatigue/high anxiety*)”, 238 (12.6%) as “high fatigue, moderate psychological symptoms (*High fatigue/moderate distress*)”, and 96 (5.1%) as overall “high symptom burden (*High*)” (Figure 1; Table 2). The *Low* profile showed normative or lower scores in all outcomes and had the lowest scores of all the identified profiles. The *Moderate fatigue/high anxiety* profile had fatigue and depressive symptoms only slightly higher than normative scores but substantially higher anxiety‐related symptoms, where anxiety‐related symptoms refer to the dimensions of worry and fear (anxiety symptoms, PTSS, and fear of recurrence). The *High fatigue/moderate distress* profile had considerably higher fatigue scores but psychological scores (depressive symptoms, anxiety symptoms, PTSS, and fear of recurrence) only slightly above normative values and the *Low* symptom burden profile. The *High* profile had the highest scores across all outcomes. Profiles with overall “low” and “high” scores emerged from the 3‐profile model onward, with little change in proportions and participant reallocation between the 3‐ and 4‐profile models (Figure 2).

**TABLE 2:** Estimated marginal means (95% CI) of the fatigue and psychological symptom outcomes for the four identified latent profiles.

Estimated proportion^a^	Low (63.3%)	Moderate fatigue/high anxiety (18.4%)	High fatigue/moderate distress (13.0%)	High (5.3%)
	Mean (95% CI)	Mean (95% CI)	Mean (95% CI)	Mean (95% CI)
**Standardized 0–100 T‐scores** ^b^				
Physical fatigue	33.0 (32.2–33.9)	42.4 (40.7–44.1)	65.8 (63.7–67.9)	74.6 (71.5–77.8)
Mental fatigue	32.1 (31.5–32.8)	36.8 (35.4–38.2)	55.4 (52.5–58.4)	66.8 (64.0–69.5)
Depressive symptoms	7.5 (6.8–8.2)	27.8 (25.9–29.6)	29.6 (27.2–32.0)	63.6 (60.2–67.0)
Anxiety symptoms	14.9 (14.1–15.7)	40.5 (38.6–42.5)	29.9 (27.5–32.3)	63.3 (60.3–66.4)
PTSS	13.1 (12.2–14.1)	48.0 (45.7–50.4)	31.7 (28.7–34.6)	63.0 (59.6–66.4)
Fear of recurrence	20.3 (19.3–21.3)	49.8 (47.5–52.1)	31.5 (28.9–34.0)	56.7 (52.6–60.7)
**Raw scores (range)**				
Physical fatigue (0–21)	6.9 (6.8–7.1)	8.9 (8.5–9.3)	13.8 (13.4–14.3)	15.7 (15.0–16.3)
Mental fatigue (0–12)	3.9 (3.8–3.9)	4.4 (4.2–4.6)	6.7 (6.3–7.0)	8.0 (7.7–8.3)
Depressive symptoms (0–15)	1.1 (1.0–1.2)	4.2 (3.9–4.4)	4.4 (4.1–4.8)	9.5 (9.0–10.1)
Anxiety symptoms (0–20)	3.0 (2.8–3.1)	8.1 (7.7–8.5)	6.0 (5.5–6.5)	12.7 (12.1–13.3)
PTSS (0–24)	3.1 (2.9–3.4)	11.5 (11.0–12.1)	7.6 (6.9–8.3)	15.1 (14.3–15.9)
Fear of recurrence (0–15)	3.0 (2.9–3.2)	7.5 (7.1–7.8)	4.7 (4.3–5.1)	8.5 (7.9–9.1)

*Note:* Questionnaires: Fatigue: Chalder's Fatigue Score; Depressive symnptoms: Patient Health Questionnaire‐9; Anxiety symptoms: The Hospital Anxiety and Depression Scale‐9, Anxiety subscale; Post‐traumatic stress symptoms PTSS: Impact of Event Scale‐6; Fear of (cancer) recurrence: Assessment of Survivor Concerns Scale.

Abbreviations: CI, confidence interval; PTSS, post‐traumatic stress symptoms.

^a^ Note that proportions are latent profile analysis estimated proportions.

^b^ All scores converted to 0–100 scales for comparability.

**TABLE 3:** Background characteristics of participants allocated to the four identified latent profiles (*N* = 1893).

	Low	Moderate fatigue/high anxiety	High fatigue/moderate distress	High	*p* ^a^
	*N* = 1216 (64.2%)	*N* = 343 (18.1%)	*N* = 238 (12.6%)	*N* = 96 (5.1%)	
**Background**					
Age at study (yrs)	43.5 (±12.1)	43.3 (±10.9)	42.2 (±11.0)	39.5 (±11.7)	0.003
Sex, female	777 (64%)	255 (74%)	172 (72%)	76 (79%)	< 0.001
Education level					0.004
Compulsory school	56 (5%)	27 (8%)	16 (7%)	12 (13%)	
Vocational school	280 (23%)	100 (29%)	55 (23%)	23 (24%)	
High school	138 (11%)	43 (13%)	32 (14%)	11 (11%)	
University	736 (61%)	171 (50%)	133 (56%)	50 (52%)	
**Cancer‐related**					
Age at diagnosis (yrs)	25.7 (±11.7)	26.7 (±11.3)	25.4 (±12.1)	23.8 (±12.9)	0.363
Time since first diagnosis (yrs)	17.1 (±6.9)	16.0 (±6.8)	16.2 (±7.0)	15.1 (±6.9)	< 0.001
Primary diagnosis group					0.003
Childhood cancer	386 (32%)	94 (27%)	80 (34%)	38 (40%)	
Melanoma	223 (18%)	45 (13%)	23 (10%)	7 (7%)	
Breast cancer	281 (23%)	107 (31%)	69 (29%)	31 (32%)	
Colorectal cancer	93 (8%)	24 (7%)	19 (8%)	7 (7%)	
Non‐Hodgkin's lymphoma	145 (12%)	44 (13%)	33 (14%)	8 (8%)	
Leukemia	88 (7%)	29 (8%)	14 (6%)	5 (5%)	
Treatment^b^					< 0.001
Minimal treatment (melanoma)	197 (17%)	40 (12%)	18 (8%)	7 (8%)	
Local treatment	142 (12%)	32 (10%)	17 (8%)	5 (5%)	
Systemic single treatment	187 (16%)	51 (16%)	33 (15%)	23 (25%)	
Multiple treatments	633 (55%)	202 (62%)	153 (69%)	56 (62%)	
Recurring or second cancer(s)	40 (3%)	9 (3%)	10 (4%)	3 (3%)	0.774

*Note:* Data are presented as mean (±SD) for continuous variables, and *n* (%) for categorical variables.

Abbreviations: SD, standard deviation; yrs, years.

^a^ *p*‐values calculated from univariable linear regression models for continuous variables and chi‐squared statistics for categorical variables.

^b^ Treatment was hierarchically aggregated into minimal treatment for melanoma (local treatment only and diagnosis of melanoma), local treatment (local radiotherapy or surgery only), systemic single treatment (including chemotherapy, stem cell transplantation and immune therapy) or multiple treatments.

Sensitivity analyses found that the identified profiles were similar whether excluding survivors of melanoma, who mainly only received minimal treatment, or stratifying by CCS and YACS (Supplement, Chapter D).

### 3.3 Survivor Characteristics Within Identified Profiles

We observed significant differences between profiles regarding age at study (*p* = 0.003), sex (*p* < 0.001), education (*p* = 0.004), time since first diagnosis (*p* < 0.001), primary diagnosis (*p* = 0.003), and treatment (*p* < 0.001; Table 3). Participants in the *Low* profile were oldest at study (43.5 years, SD 12.1), had the fewest female survivors (64%), and the highest proportion with a university degree (61%; Table 3). In contrast, those in the *High* profile were on average youngest (39.5 years, SD 11.7), had the highest proportion of female survivors (79%), and had the highest percentage with compulsory schooling only (13%). Moreover, of all profiles, the *Low* profile had the largest proportion melanoma survivors (18% vs. 7%–13%) and lowest breast cancer survivors (23% vs. 29%–32%), as well as the highest proportion who underwent minimal and local treatment (17% vs. 8%–12%). The *High* profile on the other hand had the highest proportion CCS (40% vs. 27%–34%) of all profiles, as well as fewest non‐Hodgkin's lymphoma survivors (8% vs. 12%–14%) and the most (25% vs. 15%–16%) who underwent systemic single treatments. There were no significant differences in age at diagnosis (*p* = 0.363) and recurring or second cancer(s) (*p* = 0.774) across the profiles.

Similar differences were found in sensitivity analyses, though age at study (*p* = 0.067) and primary diagnosis (*p* = 0.410) were not significantly different in the subgroup excluding melanoma survivors (Supplement, Chapter D).

### 3.4 Association Between the Profiles and Health‐Related Quality of Life

Compared to the *Low* profile, survivors allocated to the three other profiles all reported significantly lower physical and mental HRQOL (all *p* ≤ 0.001; Table 4). There were few differences in results between the univariable and multivariable models. In the multivariable models, the lowest physical and mental HRQOL scores were found in survivors allocated to the *High* profile (T‐score − 9.9, 95% CI −12.5 to −7.4 and −25.0, 95% CI −26.6 to −23.4 compared to the *Low* profile). Those in the *High* symptom burden profile and *High fatigue/moderate distress* profile (−9.4, 95% CI −10.8 to −8.1) scored similarly lower in physical HRQOL than the *Low* profile. In terms of mental HRQOL, however, those in the *High* profile scored lower than both the *Moderate fatigue/high anxiety* (−10.9, 95% CI −12.0 to −9.8) and *High fatigue/moderate distress* profiles (−11.4, 95% CI −12.6 to −10.2).

**TABLE 4:** Association between profile allocation and health‐related quality of life from univariable and multivariable linear regression models (*N* = 1814).

	Unadjusted model			Adjusted model		
	Physical HRQoL					
	*β*	95% CI	*p*	*β*	95% CI	*p*
**Latent profiles**			< 0.001			< 0.001
(1) Low	Ref.			Ref.		
(2) Moderate fatigue/high anxiety	−5.1	(−6.2 to −4.0)		−4.5	(−5.5 to −3.4)	
(3) Severely fatigued	−9.9	(−11.3 to −8.5)		−9.4	(−10.8 to −8.1)	
(4) High	−10.5	(−13.2 to −7.9)		−9.9	(−12.5 to −7.4)	

	Mental HRQoL					
	*β*	95% CI	*p*	*β*	95% CI	*p*
**Latent profiles**			< 0.001			< 0.001
(1) Low	Ref.			Ref.		
(2) Moderate fatigue/high anxiety	−10.9	(−11.9 to −9.8)		−10.9	(−12.0 to −9.8)	
(3) High fatigue/moderate distress	−11.6	(−12.8 to −10.3)		−11.4	(−12.6 to −10.2)	
(4) High	−25.4	(−27.0 to −23.8)		−25.0	(−26.6 to −23.4)	

*Note:* Covariates in adjusted models were age, sex, education level, diagnosis, treatment, time since first diagnosis, and recurring & second cancer(s). *p*‐values for overall significance are *F*‐tests, in unadjusted models, and adjusted Wald tests in adjusted models.

Abbreviations: β, beta‐coefficient; CI, confidence interval; HRQoL, health‐related quality of life; Ref., reference.

## 4 Discussion

We identified four fatigue and psychological symptom profiles from this population‐based sample of long‐term CAYACS. Two profiles showed high fatigue, one with high and another with moderate psychological symptom burden, that is, depressive symptoms, anxiety symptoms, PTSS, and fear of recurrence. Conversely, the two profiles with lower fatigue levels were also accompanied by either a higher or lower psychological symptom burden. All except the *High* symptom burden profile had depressive symptom scores near or below normative means. Survivors of the *Moderate fatigue/high anxiety* profile had a fear of recurrence score similar to the *High* profile, which had depressive symptoms scores more than double those of the other profiles.

Our findings, supported by prior studies, indicate that most CAYACS experience a (1) *Low* symptom severity burden, but a small (5%) subset of survivors experience *High* symptom burden. These two profiles were also fairly stable as the number of profiles increased, emerging early in the model with little change beyond the three‐profile model. To our knowledge, most studies in CAYACS using LPA with psychological outcomes have similarly identified four profiles [9, 16, 17, 18, 20], with a few identifying three [9, 19] or two profiles [9]. We found that 64% experienced an overall *Low* burden, similar to other papers ranging from 52% to 69% [9, 16, 17, 18, 20]. The 5% of survivors in our study with an overall *High* symptom burden also resembled these studies, ranging 5% to 14%. These distinct groups should therefore be targets for interventional strategies. The *Low* burden group might require minimal follow‐up care, with cognitive therapy for those with high anxiety‐related symptoms, and a combination of cognitive therapy and physical activity for those with high fatigue and overall high symptom burden [42].

Using distinctions in fatigue as a basis for uncovering complex relationships with psychological symptoms is supported by previous LPA/LCA studies. The US Childhood Cancer Survivor Study (CCSS), a study by Hong et al., and the Measuring Your Health study (MY‐Health) all found differences in groups of somatic and psychological symptoms [16, 19, 20]. The CCSS found that somatization and depression scores differed between two profiles, Hong et al. found consistently higher lack of energy and drowsiness scores than feelings of nervousness and sadness in all three profiles, and the MY‐Health study observed substantial differences in depression scores, yet high fatigue in two profiles excepting their overall high or low profiles. The corresponding two “middle” profiles from the St Jude Lifetime Cohort Study (SJLIFE), however, differed in their depression and anxiety scores between profiles, not fatigue [17].

Only the SJLIFE study linked symptom profiles with physical and mental HRQOL [17]. Consistent with our results, the authors found that higher symptom burden was linked to lower HRQOL, highlighting its adverse impact on well‐being. Mental HRQOL was additionally found to be more than one SD below population norms in a high depression, anxiety, and pain profile.

In line with previous literature, the *High* symptom burden profile was characterized by lower ages, more females, and a shorter time since first diagnosis [43]. Possible reasons could be greater family/work demands and more recent cancer experiences [44]. Despite similar distributions of diagnostic groups and treatment intensity across profiles, the *High* symptom burden group had proportionally more CCS, females, breast cancer survivors, and fewer cases of low treatment intensity (melanoma). Including central nervous system tumor survivors in CCS, known for their high chronic disease burden, could likely increase the *High* profile's sample size [45, 46]. Research, moreover, shows that both CCS and female survivors are more likely to experience PTSS [2]. This is reflected in a greater prevalence of breast cancer and female survivors in all burdened profiles compared to the *Low* profile.

As expected, a *High* symptom burden was associated with lower HRQOL [8, 21, 47]. In particular, fatigue appears to have a large impact on physical HRQOL, shown by low scores in both profiles with high fatigue. For mental HRQOL, both the *Moderate fatigue/high anxiety* and *High fatigue/moderate distress* profiles had similarly low levels, suggesting a similar mental burden but different manifestations—higher psychological burden in the former and greater fatigue in the latter. The *High* symptom burden profile demonstrated both high fatigue and psychological symptom severity, resulting in considerably lower relative mental HRQOL. The negative impact of fatigue and psychological symptom burden highlights the importance of addressing mental health in clinical practice, especially when fatigue symptoms may indicate complex psychological symptom patterns [1, 48].

Using LPA to identify subgroups of survivors can help understand how fatigue and psychological symptoms co‐occur at an individual level. That some individuals predominantly experience anxiety‐related symptoms with less fatigue, others experience high fatigue levels but less psychological symptom burden, and a high depressive symptoms score is more certainly accompanied by both high fatigue and anxiety‐related symptoms is useful for identifying at‐risk groups. This differentiation can aid in designing targeted interventions and follow‐up care for fatigue, anxiety symptoms, or the full spectrum of symptoms, aligning with CanCon's (Cancer Control Joint Action) emphasis on a person‐centered approach to care and tailored health plans based on survivors' risk profiles [49]. It gives ground for approaches based on each individual's situation, meeting the specific needs of high‐risk groups and developing tiered models of psychosocial care depending on symptom burden.

Our analysis suggests a critical need to identify and focus on a small subset of survivors with a *High* symptom burden as well as females, younger survivors, those with lower education levels, and more recently diagnosed survivors. Regular and early assessment of fatigue, depressive symptoms, and anxiety symptoms as recommended, could help identify survivors at risk of lower HRQOL [1]. By understanding the complexity of each survivor's unique constellation of challenges, we can move toward more holistic care to improve well‐being through targeted interventions and support. Future research should explore developing interventions, perhaps especially for high‐burden survivors, and examine opportunities for implementing early assessment of fatigue and psychological health. This could offer possibilities beyond interventions for individual conditions [43, 49, 50].

The NOR‐CAYACS study benefits from a large nationwide cohort of long‐term survivors representing a wide range of ages at diagnosis and diagnostic groups. The inclusion of questionnaires covering fatigue and multiple aspects of psychological health coupled with high‐quality data from the Cancer Registry of Norway makes this study unique in its scope and quality. We use an objective model‐based method (LPA) for determining profiles of fatigue and psychological symptoms based on multiple continuous indicators, minimizing the risk of misclassification, and allowing for better differentiation between subgroups. We maintain confidence in the robustness of our profiles based on clinical interpretability, concordance with previous studies, a ≥ 5% cutoff, robustness across sensitivity analyses, high entropy, and high posterior probabilities. One limitation of our study was the low response rate of 42%, but a previous paper of the NOR‐CAYACS study showed little evidence for non‐response bias [23]. Generalizability is moreover limited by an overrepresentation of females (73%) among young adult survivors, mainly due to a large number of breast cancer survivors and female melanoma survivors. However, sensitivity analyses showed that the four profiles remained robust across subgroups.

## 5 Conclusion

Our study found four symptom burden profiles, two with high fatigue but differing psychological symptom burden, one with normative fatigue levels but high psychological symptom burden, and one overall low symptom burden profile. These profiles provide insight for identifying at‐risk individuals and the development of effective, holistic, person‐centered strategies in CAYACS. LPA allows for multimodal interventions targeting survivors' unique constellation of symptom burden simultaneously, moving beyond interventions for individual conditions.

## Author Contributions

**Wei H. Deng:** data curation (equal), formal analysis (lead), methodology (equal), validation (equal), visualization (lead), writing – original draft (lead), writing – review and editing (lead). **Hanne C. Lie:** conceptualization (equal), data curation (equal), funding acquisition (equal), investigation (equal), project administration (equal), resources (equal), supervision (equal), validation (equal), writing – review and editing (equal). **Ellen Ruud:** data curation (equal), resources (equal), validation (equal), writing – review and editing (equal). **Jon H. Loge:** data curation (equal), funding acquisition (equal), investigation (equal), project administration (equal), resources (equal), validation (equal), writing – review and editing (equal). **Cecilie E. Kiserud:** data curation (equal), funding acquisition (equal), investigation (equal), project administration (equal), resources (equal), supervision (equal), validation (equal), writing – review and editing (equal). **Corina S. Rueegg:** conceptualization (equal), data curation (equal), formal analysis (equal), funding acquisition (equal), methodology (equal), resources (equal), software (equal), supervision (lead), validation (equal), visualization (equal), writing – original draft (equal), writing – review and editing (equal).

## Ethics Statement and Consent to Participate

The study was performed in accordance with the Declaration of Helsinki and approved by the Regional Committee for Medical Research Ethics (2015/232), the Norwegian Data Protection Authority (15/00395‐2/CGN), and the Norwegian Cancer Registry and the Data Protection Officer at Oslo University. Written informed consent was obtained for both participation in the survey and data linkage to information at the Cancer Registry of Norway. Data protection is assured by pseudonymization.

## Conflicts of Interest

The authors declare no conflicts of interest.

## Supporting information


Data S1.

## Data Availability

De‐identified individual participant data that underlie the results reported in this article is available from HCL, the project leader, upon reasonable request and from the time point of publication of the article.
