# Supplementary material for: Profiles of Fatigue and Psychological Symptoms in Long‐Term Childhood, Adolescent, and Young Adult Cancer Survivors—The NOR‐CAYACS Study
Source: Cancer Med. 2024 Nov 25;13(22):e70425. doi: 10.1002/cam4.70425 (PMC11586778; doi:10.1002/cam4.70425)
Supplement: Supplementary file 1 — Data S1. [file CAM4-13-e70425-s001.docx]

# Profiles of fatigue and psychological symptoms in long-term childhood, adolescent, and young adult cancer survivors – The NOR-CAYACS Study

**Wei H Deng^1,2^, Hanne C Lie^3,4^, Ellen Ruud^5,6^, Jon H Loge^4^, Cecilie E Kiserud^3^, Corina S Rueegg^1,7^**

^1^ Oslo Centre for Biostatistics and Epidemiology, Oslo University Hospital, Oslo, Norway

^2^ Oslo Centre for Biostatistics and Epidemiology, Department of Biostatistics, Institute of Basic Medical Sciences, University of Oslo, Oslo, Norway

^3^ National Advisory Unit on Late Effects after Cancer Treatment, Oslo University Hospital, Oslo, Norway

^4^ Department of Behavioral Medicine, Institute of Basic Medical Sciences, University of Oslo, Oslo, Norway

^5^ Department of Paediatric Haematology and Oncology, Division for Paediatric and Adolescence Medicine, Oslo University Hospital, Oslo, Norway

^6^ Institute for Clinical Medicine, Faculty of Medicine, University of Oslo, Oslo, Norway

^7^ Epidemiology, Biostatistics and Prevention Institute, University of Zurich, Zurich, Switzerland

Table of Contents

[Profiles of fatigue and psychological symptoms in long-term childhood, adolescent, and young adult cancer survivors – The NOR-CAYACS Study 1](#_Toc165157709)

[A. Supplementary methods 4](#_Toc165157710)

[Supplemental figure 1: Sample selection flowchart 4](#_Toc165157711)

[Supplemental figure 2: Directed acyclic graph (DAG) of relationships between fatigue and psychological symptom profiles and health-related quality of life 5](#_Toc165157712)

[Standardized T-scores 6](#_Toc165157713)

[Normative values for fatigue and psychological symptoms 6](#_Toc165157714)

[Latent profile analysis: Fit indices 6](#_Toc165157715)

[B. Fatigue and psychological symptom outcomes 8](#_Toc165157716)

[Supplemental table 1: Fatigue and psychological symptom outcomes overall and by age group at diagnosis and diagnosis, mean (SD) 8](#_Toc165157717)

[C. Latent profile analysis results 9](#_Toc165157718)

[Supplemental table 2: Fit statistics and classification coefficients for all latent profile models 9](#_Toc165157719)

[Supplemental table 3: Average posterior probability matrix for the four-profile latent profile analysis model 10](#_Toc165157720)

[D. Sensitivity analyses 11](#_Toc165157721)

[Sensitivity analysis 1: Latent profile analysis and related characteristics excluding melanoma survivors 11](#_Toc165157722)

[Supplemental figure 3: (Melanoma excluded) Estimated marginal mean T-scores for fatigue and psychological symptom burdens for the four identified latent profiles (N=1595) 11](#_Toc165157723)

[Supplemental table 4: (Melanoma excluded) Estimated marginal means (95%CI) for fatigue and psychological symptom burdens for the four identified latent profiles 12](#_Toc165157724)

[Supplemental table 5: (Melanoma excluded) Fit statistics and classification coefficients for all latent profile models 13](#_Toc165157725)

[Supplemental table 6: (Melanoma excluded) Background characteristics of participants allocated to the four identified latent profiles (N=1595) 14](#_Toc165157726)

[Sensitivity analysis 2: Latent profile analysis and related characteristics in childhood cancer survivors only 15](#_Toc165157727)

[Supplemental figure 4: (Childhood cancer survivors only) Estimated marginal mean T-scores for fatigue and psychological symptom burdens for the four identified latent profiles (N=598) 15](#_Toc165157728)

[Supplemental table 7: (Childhood cancer survivors only) Estimated marginal means (95%CI) for fatigue and psychological symptom burdens for the four identified latent profiles 16](#_Toc165157729)

[Supplemental table 8: (Childhood cancer survivors only) Fit statistics and classification coefficients for all latent profile models 17](#_Toc165157730)

[Supplemental table 9: (Childhood cancer survivors only) Background characteristics of participants allocated to the four identified latent profiles (N=598) 18](#_Toc165157731)

[Sensitivity analysis 3: Latent profile analysis and related characteristics in young adult cancer survivors only 19](#_Toc165157732)

[Supplemental figure 5: (Young adult survivors only) Estimated marginal mean T-scores for fatigue and psychological symptom burdens for the four identified latent profiles (N=1295) 19](#_Toc165157733)

[Supplemental table 10: (Young adult survivors only) Estimated marginal means (95%CI) for fatigue and psychological symptom burdens for the four identified latent profiles 20](#_Toc165157734)

[Supplemental table 11: (Young adult survivors only) Fit statistics and classification coefficients for all latent profile models 21](#_Toc165157735)

[Supplemental table 12: (Young adult survivors only) Background characteristics of participants allocated to the four identified latent profiles (N=1295) 22](#_Toc165157736)

[References 23](#_Toc165157737)

# Supplementary methods

Supplemental figure 1: Sample selection flowchart


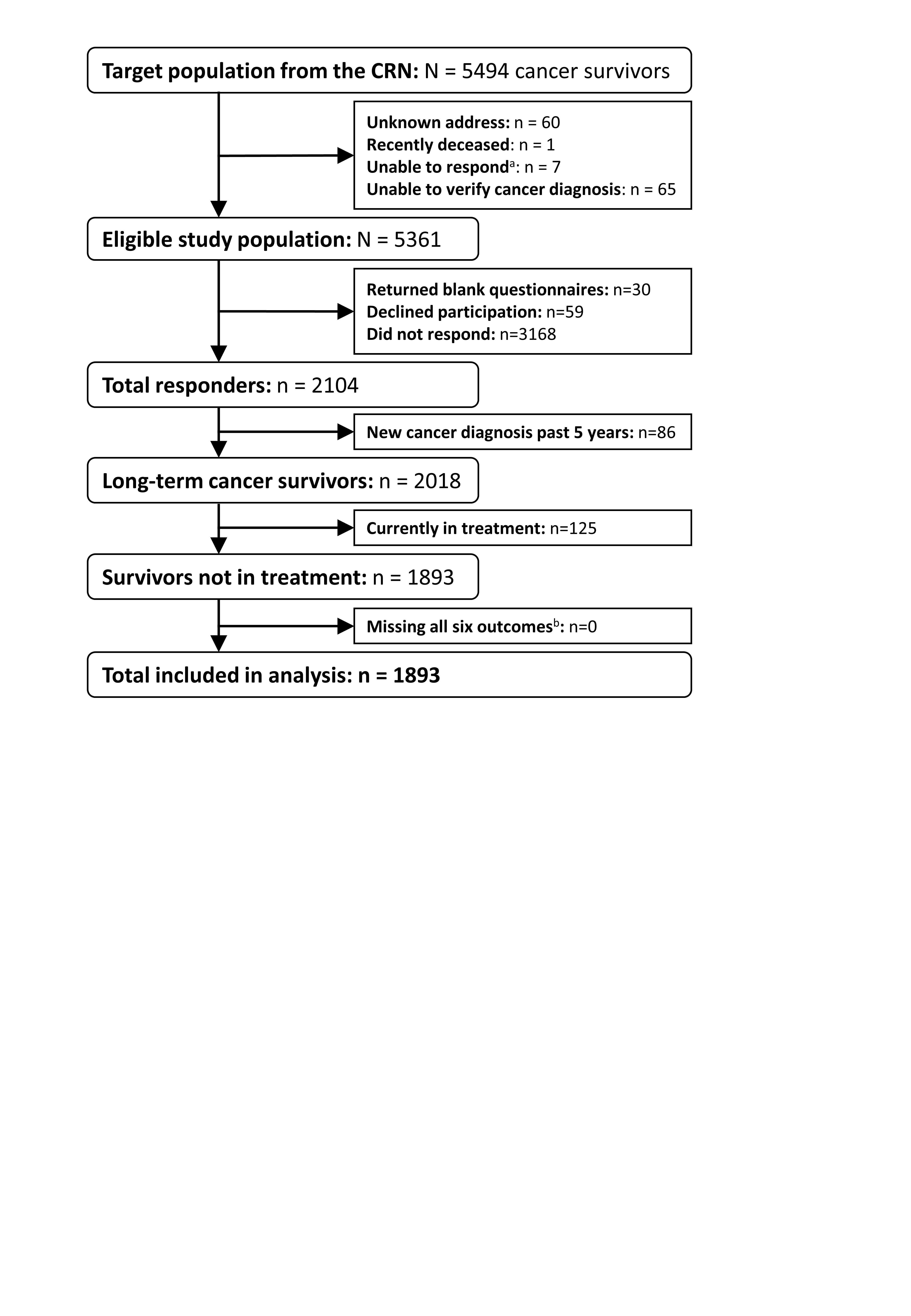


CRN: Cancer Registry of Norway; n: number of survivors.
^a^ Due to cognitive limitations.
^b^ Missing response on all physical and mental fatigue, depressive symptoms, anxiety symptoms, post-traumatic stress symptoms, and fear of recurrence scores.

Supplemental figure 2: Directed acyclic graph (DAG) of relationships between fatigue and psychological symptom profiles and health-related quality of life


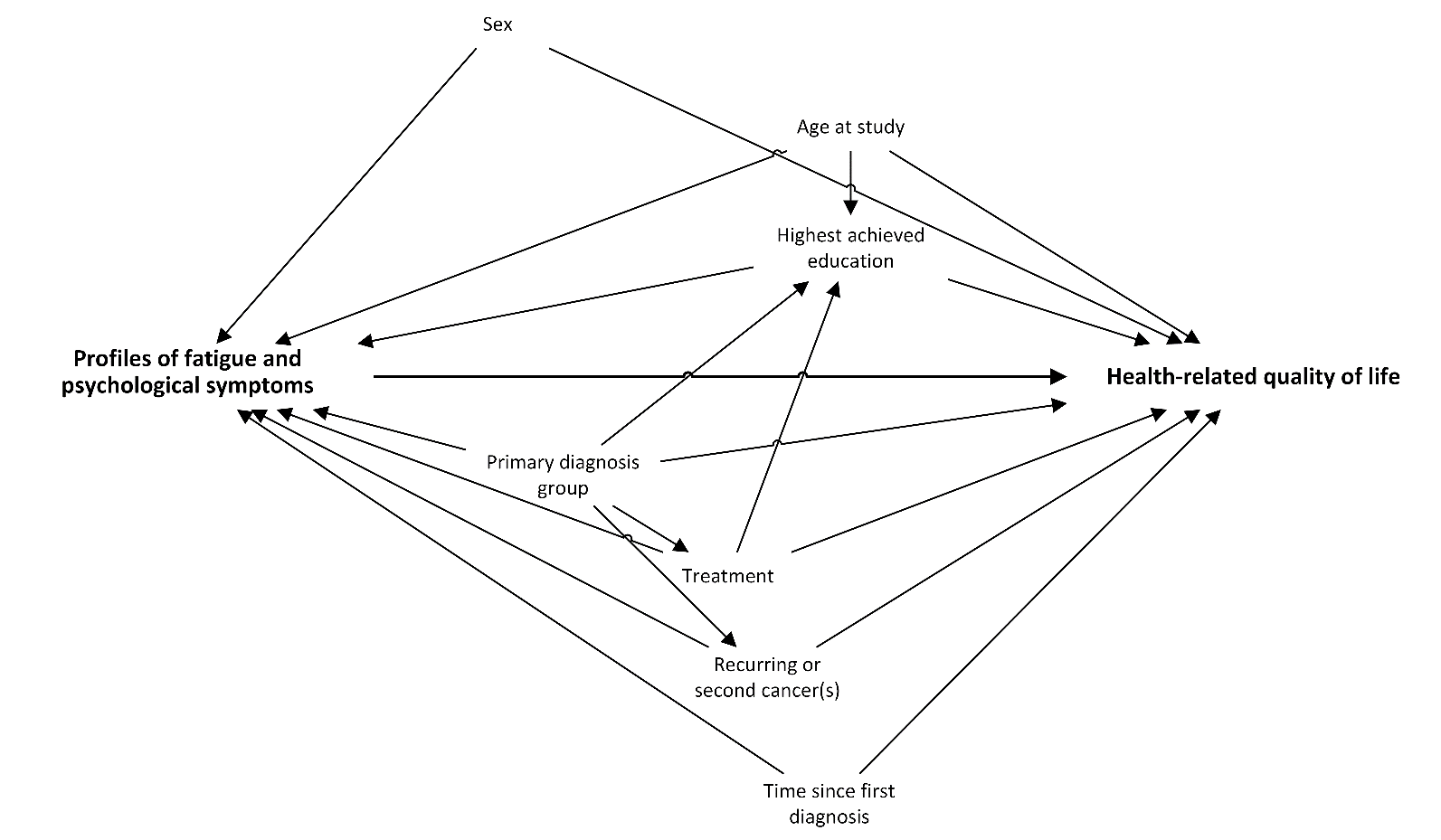


Supplemental figure 1 represents the relationship between the exposure “profiles of fatigue and psychological symptoms” and outcome “health-related quality of life” and covariates in their causal path. Minimal sufficient adjustment set for estimating the total effect are age at study, sex, highest achieved education, primary (cancer) diagnosis group, (cancer) treatment, time since first diagnosis, and recurring & second cancer(s).

## Standardized T-scores

All outcome scales were standardized to T-scores ranging from 0 to 100 with the following method:

$$\text{T-score}=\frac{x-min(x)}{\max\left( x \right)-\min(x)}*100$$

Where x = outcome measure, min/max = lowest/highest possible score of each outcome.

## Normative values for fatigue and psychological symptoms

Norwegian reference populations were identified through literature searches and chosen based on quality of data, sample size, and comparability to our sample demographic. We present normative scores for fatigue, depressive symptoms, and anxiety symptoms.^1,2^ There are no normative values for PTSS and fear of recurrence. Mean mental and physical fatigue scores were calculated from sex and age stratified reference data from 2015, aged 18-80 years (n=2041).^1^ A mean normative PHQ-9 depressive symptoms score was calculated based on sex stratified data from the same reference population (n=2086).^1^ Normative scores for anxiety symptoms were from sex stratified data of 52769 individuals aged 20-89 years, collected between 1995 and 1997.^2^ All normative scores were likewise standardized to range 0-100.

## Latent profile analysis: Fit indices

We used latent profile analysis (LPA) to investigate the presence of unobserved latent subgroups in our sample of childhood, adolescent, and young adult cancer survivors (CAYACs). The goal of using LPA in this population was to identify meaningful groups of survivors based on within-group similarities of our chosen fatigue and psychological symptom outcomes. This approach is model based (data driven) and avoids the use of arbitrary cut-offs to define binary variables as opposed to for example latent cluster analysis. It therefore offers a mathematical evaluation of how the model fits the underlying data.

The Bayesian information criterion (BIC) was chosen as one of the primary fit indices based on previous published research.^3,4^ Nylund et al. (2007) found that it was the most consistent in identifying the correct number of profiles in models with continuous outcomes.^3^ It is also one of the recommended fit indices in the Guidelines for Reporting on Latent Trajectory Studies (GRoLTS).^5^ The formula for BIC is given by: BIC = k*ln(n)-2ln(θ). Where *k* is the number of profiles being tested, n the sample size, and θ the maximum likelihood of the current model. Lower values indicate better fit. The BIC therefore penalizes *k* higher number of profiles and favors models with lower number of profiles.

Entropy is a measure of degree of separation between each latent profile, where higher values indicate better separation (more distinct groups).^4^ It gives an indication of the quality of distinction for each model.^6^ It provides a number between 0 and 1; the closer to 1 the better discrimination/classification.

We decided that the lowest model estimated profile proportion to be ≥5% as a rough estimate for detecting a clinically relevant sample.

The LPA provides for each subject the estimated posterior probability for belonging to each profile. We estimated for each individual this probability of profile membership for each profile in the four-profile model.

We also present often used indices Akaike information criterion (AIC), a modified Bayes factor (BF), and Vuong-Lo-Mendell-Rubin likelihood-ratio test (VLMR-LRT) in Supplemental table 2 for completeness.^7,8^ AIC has been shown to give naive results and sometimes overestimates the number of profiles as it does not penalize larger models.^3^ Bayes factor^9^ and the VLMR-LRT^7^ tests compares fit between current model *k* and *k*+1 or *k*-1 models respectively. These test whether a model is significantly better than the latter. A non-significant result means that no improvement was observed for the current *k*^th^ model.

## Sensitivity analyses

To investigate the robustness of our models, we performed sensitivity analyses in three subgroups. Excluding melanoma survivors allowed us to investigate how the model was affected by removing this distinct group of survivors who primarily underwent minimal treatment (Table 1) and are considered to have fewer late effects. Restricting our sample to just childhood cancer survivors (CCS) or young adult cancer survivors (YACS) would further inform whether the same or different profiles exist depending on the age of cancer diagnosis. These groups have distinctive characteristics and cancer types, and we therefore provide results separately for readers interested in each of these cancer groups.

# Fatigue and psychological symptom outcomes

Supplemental table 1: Fatigue and psychological symptom outcomes overall and by age group at diagnosis and diagnosis, mean (SD)

|  | Age group at diagnosis | | Diagnosis (young adult cancer survivors) | | | | |  |
| --- | --- | --- | --- | --- | --- | --- | --- | --- |
|  | **Childhood cancer** | **Young adult cancer** | **Melanoma** | **Breast Cancer** | **Colorectal** | **NHL** | **Leukemia** | **Total** |
|  | **N=598** | **N=1295** | **N=298** | **N=488** | **N=143** | **N=230** | **N=136** | **N=1893** |
| **Standardized 0-100 T-scores^a^** |  |  |  |  |  |  |  |  |
| Physical fatigue | 42.0 (19.4) | 40.9 (17.7) | 37.7 (15.8) | 42.8 (18.3) | 41.3 (19.6) | 41.7 (17.0) | 39.1 (17.8) | 41.3 (18.3) |
| Mental fatigue | 37.5 (16.5) | 38.1 (14.4) | 35.4 (13.0) | 39.6 (14.8) | 37.9 (16.6) | 39.2 (13.5) | 36.5 (14.1) | 37.9 (15.1) |
| Depressive symptoms | 19.2 (20.5) | 16.1 (17.8) | 12.0 (15.3) | 18.5 (19.0) | 16.4 (18.8) | 16.1 (16.2) | 15.9 (18.1) | 17.1 (18.7) |
| Anxiety symptoms | 26.3 (20.3) | 23.1 (18.3) | 21.0 (17.0) | 24.7 (19.1) | 21.3 (17.4) | 23.5 (18.6) | 23.5 (18.1) | 24.2 (19.0) |
| PTSS | 23.2 (23.0) | 25.1 (21.7) | 17.9 (18.6) | 28.2 (21.8) | 24.9 (22.7) | 27.7 (22.0) | 25.4 (22.3) | 24.5 (22.1) |
| Fear of recurrence | 26.5 (22.6) | 30.2 (20.4) | 29.2 (19.1) | 33.0 (20.9) | 27.6 (20.9) | 29.7 (20.1) | 26.1 (20.1) | 29.0 (21.2) |
| **Raw scores (range)** |  |  |  |  |  |  |  |  |
| Physical fatigue (0-21) | 8.8 (4.1) | 8.6 (3.7) | 7.9 (3.3) | 9.0 (3.8) | 8.7 (4.1) | 8.7 (3.6) | 8.2 (3.7) | 8.7 (3.8) |
| Mental fatigue (0-12) | 4.5 (2.0) | 4.6 (1.7) | 4.2 (1.6) | 4.8 (1.8) | 4.6 (2.0) | 4.7 (1.6) | 4.4 (1.7) | 4.5 (1.8) |
| Depressive symptoms (0-15) | 2.9 (3.1) | 2.4 (2.7) | 1.8 (2.3) | 2.8 (2.9) | 2.5 (2.8) | 2.4 (2.4) | 2.4 (2.7) | 2.6 (2.8) |
| Anxiety symptoms (0-20) | 5.3 (4.1) | 4.6 (3.7) | 4.2 (3.4) | 4.9 (3.8) | 4.3 (3.5) | 4.7 (3.7) | 4.7 (3.6) | 4.8 (3.8) |
| PTSS (0-24) | 5.6 (5.5) | 6.0 (5.2) | 4.3 (4.5) | 6.8 (5.2) | 6.0 (5.4) | 6.7 (5.3) | 6.1 (5.4) | 5.9 (5.3) |
| Fear of recurrence (0-15) | 4.0 (3.4) | 4.5 (3.1) | 4.4 (2.9) | 4.9 (3.1) | 4.1 (3.1) | 4.5 (3.0) | 3.9 (3.0) | 4.4 (3.2) |

^a^ All scores converted to 0-100 scales for comparability.
Childhood cancers are ages up to and including 18 years, young adult cancers are from 19 to 39 years.
Questionnaires: Fatigue: Chalder’s Fatigue Score; Depressive symptoms: Patient Health Questionnaire-9; Anxiety symptoms: The Hospital Anxiety and Depression Scale-9, Anxiety subscale; PTSS: Impact of Event Scale-6; Fear of (cancer) recurrence: Assessment of Survivor Concerns Scale.
Abbreviations: NHL: non-Hodgkin’s lymphoma, SD: standard deviation, N: number, PTSS: post-traumatic stress symptoms.

# Latent profile analysis results

Supplemental table 2: Fit statistics and classification coefficients for all latent profile models

| K | LL | AIC | BIC | BF | VLMR-LRT | Entropy | PPmax | est.%min |
| --- | --- | --- | --- | --- | --- | --- | --- | --- |
| 1 | -29139 | 58301.14 | 58368 | 0 | - | - | 1.00 (0.00) | 100.0 % |
| 2 | -27429 | 54895 | 55001 | <0.001 | 0 | **0.891** | 0.97 (0.09) | 26.2% |
| 3 | -26951 | 53955 | 54099 | <0.001 | <0.001 | 0.877 | 0.94 (0.11) | 6.9% |
| 4 | -26701 | 53467 | 53651 | <0.001 | <0.001 | 0.883 | 0.94 (0.13) | **5.3%** |
| 5 | -26577 | 53234 | 53456 | <0.001 | <0.001 | 0.818 | 0.88 (0.15) | 4.2% |
| 6 | -26469 | 53032 | 53292 | <0.001 | <0.001 | 0.842 | 0.88 (0.15) | 4.1% |
| 7 | **-26301** | **52710** | **53010** |  | <0.001 | 0.881 | 0.91 (0.14) | 3.0% |

Note: Bold values indicate “best” fit.

Abbreviations: AIC = Aikaike information criterion; BF = Bayes factor; BIC = Bayesian information criterion; est.%_min_ = model estimated proportion of participants in the smallest class; K = number of classes; LL = log-likelihood; PP_max_ = mean propensity probability of the single profile with highest probability (where participants were allocated by modal assignment); VLMR-LRT = Vuong-Lo-Mendell-Rubin adjusted likelihood-ratio-test.

We chose the four-profile model (highlighted yellow) based on clinical interpretability and statistical fit. Bayesian information criterion (BIC) did not indicate any best fit, showing better fit with increasing number of profiles through to seven. However, we observed a smaller change of improvement from four profiles upwards, suggesting largest improvements were found up to the four-profile model. Though the two-profile model had highest entropy, where higher values up to 1.00 denote better profile separation, no favored model emerged as all entropies were high and differed little (range 0.82-0.89). The highest number of profiles with estimated proportion in the smallest single profile still ≥5% was the four-profile model (5.3%).

Supplemental table 3: Average posterior probability matrix for the four-profile latent profile analysis model

| Profile | 1 | 2 | 3 | 4 |
| --- | --- | --- | --- | --- |
| 1. Low | **0.962** | 0.052 | 0.042 | 0.000 |
| 2. Moderate fatigue/high anxiety | 0.022 | **0.883** | 0.076 | 0.016 |
| 3. High fatigue/moderate distress | 0.016 | 0.053 | **0.864** | 0.028 |
| 4. Comorbid | 0.000 | 0.013 | 0.018 | **0.956** |

Note: Average probability of profile membership (column) by latent profile modal assignment (row) in the NOR-CAYACS study sample. Diagonal bold values are average posterior probabilities of assigned class. The closer diagonal elements are to 1 and the closer the off-diagonal elements are to 0, the better the classification/entropy.

# Sensitivity analyses

We present here results from the sensitivity analyses for our LPA and description of background characteristics of identified profiles. All starting values and chosen continuous outcomes were kept the same as the primary model, only changing the sample accordingly: 1) excluding melanoma survivors, 2) childhood cancer survivors only, and 3) young adult cancer survivors only. These were chosen to check for robustness of our model across the most common survivor groupings.

Sensitivity analyses show similar patterns for all outcomes across all three sensitivity analyses. BIC improved with increasing number of profiles and entropy was high across all models (0.82-0.91). A notable difference that the three-profile model had a lowest estimated proportion of a single profile of at least 5% in both the 2) CCS only and 3) YACS only analyses. The four-profile models were however very close at 4.8% and 4.9% respectively. Below, we present marginal means and characteristics of the four-profile models for comparison to our primary analysis.

## Sensitivity analysis 1: Latent profile analysis and related characteristics excluding melanoma survivors

Supplemental figure 3: (Melanoma excluded) Estimated marginal mean T-scores for fatigue and psychological symptom burdens for the four identified latent profiles (N=1595)


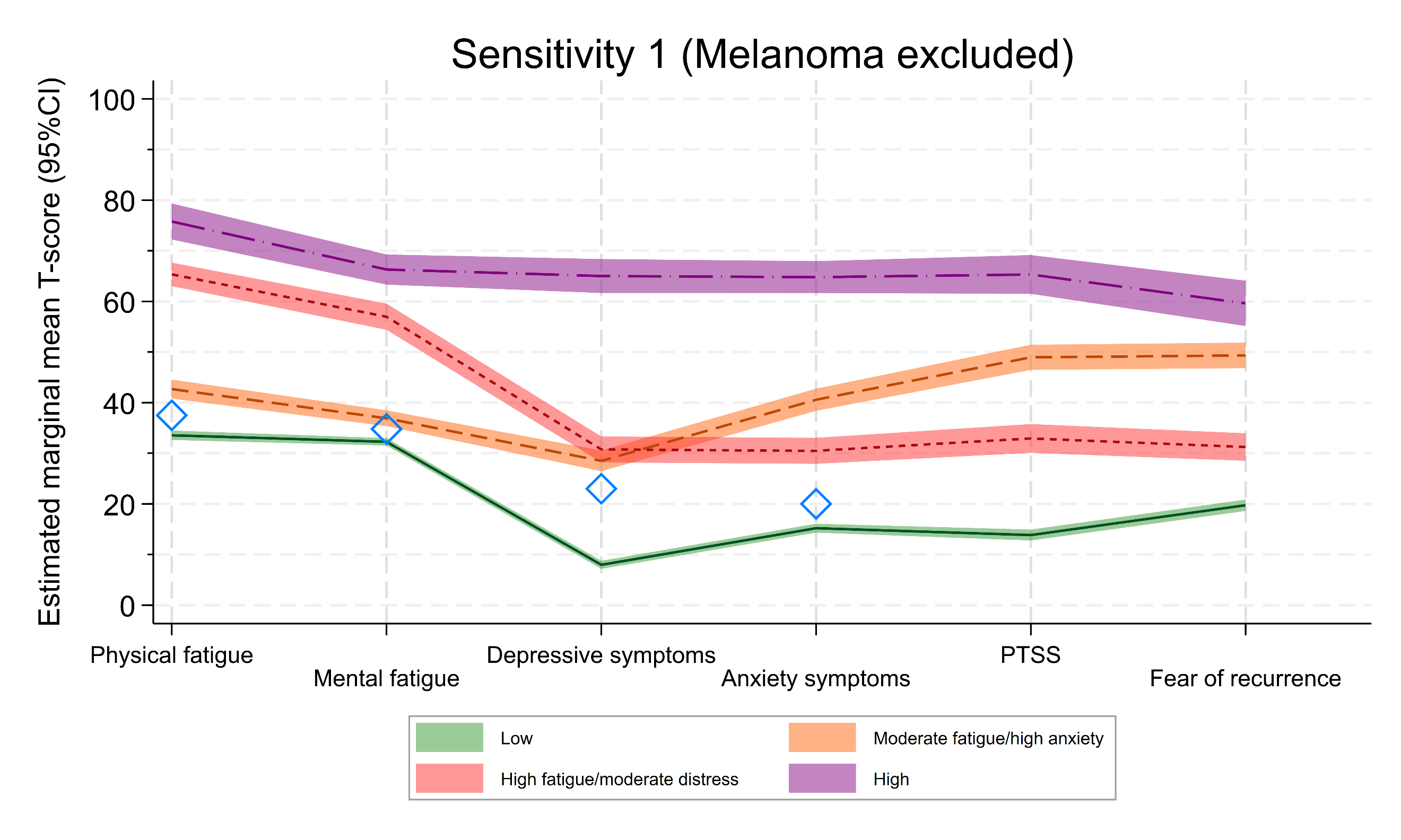


The figure shows results from the latent profile analysis where melanoma survivors were excluded with marginal mean T-scores (darker solid and dashed lines) with 95% confidence intervals (light colored areas) for each of the four identified profiles. T-score is a standardized scale from 0 to 100 for all outcomes.

**◇** Normative scores for fatigue, depressive symptoms, and anxiety symptoms from Norwegian populations ^1,2^Abbreviations: PTSS: post-traumatic stress symptoms.

Supplemental table 4: (Melanoma excluded) Estimated marginal means (95%CI) for fatigue and psychological symptom burdens for the four identified latent profiles

| Estimated proportion^a^ | Low  (62.1%) | Moderate fatigue/ high anxiety (19.0%) | High fatigue/ moderate distress (13.6%) | High  (5.4%) |
| --- | --- | --- | --- | --- |
|  | Mean (95% CI) | Mean (95% CI) | Mean (95% CI) | Mean (95% CI) |
| Standardized 0-100 T-scores^b^ |  |  |  |  |
| Physical fatigue | 33.6 (32.6-34.5) | 42.7 (40.8-44.6) | 65.3 (63.0-67.7) | 75.8 (72.2-79.3) |
| Mental fatigue | 32.3 (31.5-33.0) | 36.9 (35.3-38.5) | 57.0 (54.4-59.6) | 66.3 (63.3-69.3) |
| Depressive symptoms | 8.0 (7.2-8.8) | 28.5 (26.5-30.6) | 30.8 (28.2-33.3) | 65.0 (61.7-68.4) |
| Anxiety symptoms | 15.2 (14.3-16.1) | 40.6 (38.4-42.8) | 30.5 (27.9-33.1) | 64.8 (61.6-67.9) |
| PTSD | 13.8 (12.8-14.9) | 49.0 (46.5-51.4) | 32.9 (30.1-35.8) | 65.3 (61.5-69.1) |
| Fear of recurrence | 19.7 (18.6-20.9) | 49.3 (46.8-51.9) | 31.2 (28.5-34.0) | 59.6 (55.1-64.1) |
| Raw scores (range) |  |  |  |  |
| Physical fatigue (0-21) | 7.0 (6.9-7.2) | 9.0 (8.6-9.4) | 13.7 (13.2-14.2) | 15.9 (15.2-16.7) |
| Mental fatigue (0-12) | 3.9 (3.8-4.0) | 4.4 (4.2-4.6) | 6.8 (6.5-7.1) | 8.0 (7.6-8.3) |
| Depressive symptoms (0-15) | 1.2 (1.1-1.3) | 4.3 (4.0-4.6) | 4.6 (4.2-5.0) | 9.8 (9.2-10.3) |
| Anxiety symptoms (0-20) | 3.0 (2.9-3.2) | 8.1 (7.7-8.6) | 6.1 (5.6-6.6) | 13.0 (12.3-13.6) |
| PTSD (0-24) | 3.3 (3.1-3.6) | 11.7 (11.2-12.3) | 7.9 (7.2-8.6) | 15.7 (14.8-16.6) |
| Fear of recurrence (0-15) | 3.0 (2.8-3.1) | 7.4 (7.0-7.8) | 4.7 (4.3-5.1) | 8.9 (8.3-9.6) |

^a^ Proportions are latent profile analysis estimated proportions.
^b^ All scores converted to 0-100 scales for comparability.
Questionnaires: Fatigue: Chalder’s Fatigue Score; Depressive symptoms: Patient Health Questionnaire-9; Anxiety symptoms: The Hospital Anxiety and Depression Scale-9, Anxiety subscale; PTSS: Impact of Event Scale-6; Fear of (cancer) recurrence: Assessment of Survivor Concerns Scale.

Abbreviations: CI: confidence interval.

Supplemental table 5: (Melanoma excluded) Fit statistics and classification coefficients for all latent profile models

| K | LL | AIC | BIC | BF | VLMR-LRT | Entropy | PPmax | est.%min |
| --- | --- | --- | --- | --- | --- | --- | --- | --- |
| 1 | -24773 | 49570 | 49634 | 0.000 | - | - | 1.00 (0.00) | 100.0% |
| 2 | -23343 | 46725 | 46827 | <0.001 | 0 | 0.886 | 0.97 (0.09) | 26.9% |
| 3 | -22935 | 45921 | 46061 | <0.001 | <0.001 | 0.879 | 0.94 (0.11) | 6.5% |
| 4 | -22724 | 45514 | 45691 | <0.001 | <0.001 | 0.880 | 0.93 (0.12) | 5.4% |
| 5 | -22632 | 45343 | 45558 | <0.001 | <0.001 | 0.803 | 0.87 (0.15) | 4.4% |
| 6 | -22514 | 45121 | 45374 | <0.001 | <0.001 | **0.888** | 0.92 (0.13) | 4.2% |
| 7 | **-22395** | **44898** | **45189** |  | <0.001 | 0.875 | 0.91 (0.15) | 3.4% |

Note: Bold values indicate “best” fit.

Abbreviations: AIC = Aikaike information criterion; BF = Bayes factor; BIC = Bayesian information criterion; est.%_min_ = model estimated proportion of participants in the smallest class; K = number of classes; LL = log-likelihood; PP_max_ = mean propensity probability of the single profile with highest probability (where participants were allocated by modal assignment); VLMR-LRT = Vuong-Lo-Mendell-Rubin adjusted likelihood-ratio-test.

Supplemental table 6: (Melanoma excluded) Background characteristics of participants allocated to the four identified latent profiles (N=1595)

|  | Low | Moderate fatigue/  high anxiety | High fatigue/  moderate distress | High | p-value^a^ |
| --- | --- | --- | --- | --- | --- |
|  | N=1006  (63.1%) | N=293  (18.4%) | N=212  (13.3%) | N=84  (5.3%) |  |
| Background |  |  |  |  |  |
| Age at study (yrs) | 42.1 (12.4) | 42.3 (10.8) | 41.4 (11.0) | 39.1 (12.1) | 0.067 |
| Sex, female | 629 (63%) | 220 (75%) | 150 (71%) | 68 (81%) | <0.001 |
| Highest achieved education |  |  |  |  | 0.005 |
| Compulsory school | 50 (5%) | 20 (7%) | 16 (8%) | 12 (14%) |  |
| Vocational school | 234 (23%) | 88 (30%) | 46 (22%) | 21 (25%) |  |
| High school | 116 (12%) | 39 (13%) | 29 (14%) | 9 (11%) |  |
| University | 602 (60%) | 144 (49%) | 119 (57%) | 42 (50%) |  |
| Cancer related |  |  |  |  |  |
| Age at diagnosis (yrs) | 24.4 (±12.3) | 25.9 (±11.8) | 25.1 (±12.3) | 23.3 (±13.2) | 0.799 |
| Time since first diagnosis (yrs) | 17.1 (±6.9) | 15.8 (±6.6) | 15.8 (±7.1) | 15.2 (±7.0) | <0.001 |
| Primary diagnosis group |  |  |  |  | 0.410 |
| Childhood cancer | 392 (39%) | 94 (32%) | 78 (37%) | 34 (40%) |  |
| Breast cancer | 285 (28%) | 104 (35%) | 69 (33%) | 30 (36%) |  |
| Colorectal cancer | 94 (9%) | 24 (8%) | 18 (8%) | 7 (8%) |  |
| Non-Hodgkin's lymphoma | 147 (15%) | 42 (14%) | 33 (16%) | 8 (10%) |  |
| Acute lymphatic leukemia | 88 (9%) | 29 (10%) | 14 (7%) | 5 (6%) |  |
| Treatment^b^ |  |  |  |  | 0.012 |
| Local treatment | 144 (15%) | 32 (12%) | 15 (8%) | 5 (6%) |  |
| Systemic single treatment | 182 (19%) | 48 (17%) | 34 (17%) | 21 (27%) |  |
| Multiple treatments | 633 (66%) | 198 (71%) | 149 (75%) | 53 (67%) |  |
| Recurring or second cancer(s) | 33 (3%) | 7 (2%) | 10 (5%) | 3 (4%) | 0.550 |

^a^ p-values calculated from univariable linear regression models for continuous variables and chi-squared statistics for categorical variables.
^b^ Treatment was hierarchically aggregated into minimal treatment for melanoma (local treatment only and diagnosis of melanoma), local treatment (local radiotherapy or surgery only), systemic single treatment (including chemotherapy, stem cell transplantation and immune therapy) or multiple treatments.
Data are presented as mean (±SD) for continuous variables, and n (%) for categorical variables.

Abbreviations: SD: standard deviation; yrs: years.

## Sensitivity analysis 2: Latent profile analysis and related characteristics in childhood cancer survivors only

Supplemental figure 4: (Childhood cancer survivors only) Estimated marginal mean T-scores for fatigue and psychological symptom burdens for the four identified latent profiles (N=598)


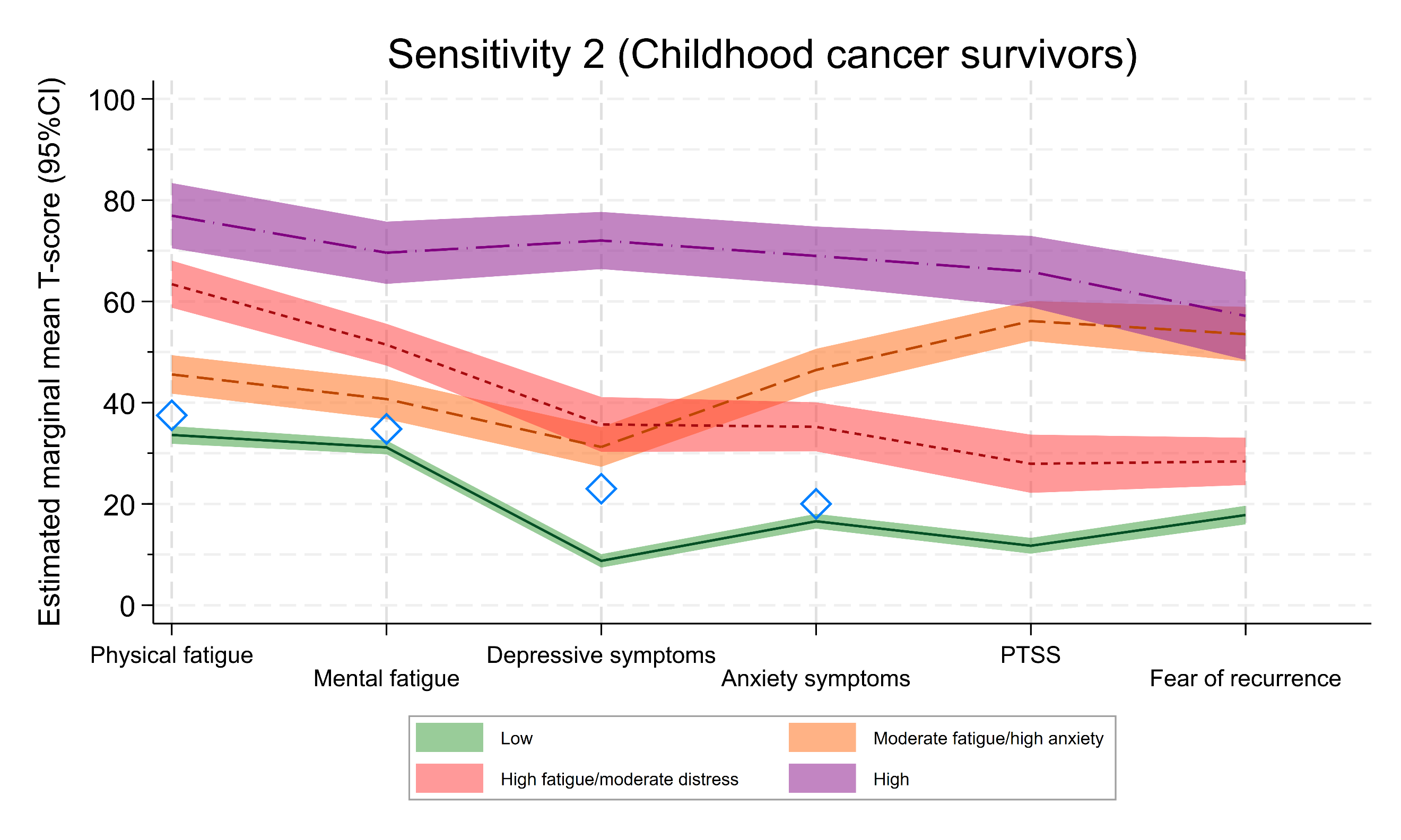


The figure shows results from the latent profile analysis where childhood cancer survivors were included with marginal mean T-scores (darker solid and dashed lines) with 95% confidence intervals (light colored areas) for each of the four identified profiles. T-score is a standardized scale from 0 to 100 for all outcomes.

**◇** Normative scores for fatigue, depressive symptoms, and anxiety symptoms from Norwegian populations ^1,2^

Abbreviations: PTSS: post-traumatic stress symptoms.

Supplemental table 7: (Childhood cancer survivors only) Estimated marginal means (95%CI) for fatigue and psychological symptom burdens for the four identified latent profiles

| Estimated proportion^a^ | Low  (65.2%) | Moderate fatigue/ high anxiety (15.1%) | High fatigue/ moderate distress (15.0%) | High  (4.8%) |
| --- | --- | --- | --- | --- |
|  | Mean (95% CI) | Mean (95% CI) | Mean (95% CI) | Mean (95% CI) |
| Standardized 0-100 T-scores^b^ |  |  |  |  |
| Physical fatigue | 33.6 (31.9-35.4) | 63.4 (58.7-68.0) | 45.6 (41.8-49.4) | 76.9 (70.5-83.4) |
| Mental fatigue | 31.2 (29.8-32.5) | 51.4 (47.3-55.6) | 40.7 (36.7-44.7) | 69.6 (63.5-75.8) |
| Depressive symptoms | 8.8 (7.4-10.1) | 35.7 (30.3-41.1) | 31.2 (27.3-35.2) | 72.0 (66.4-77.7) |
| Anxiety symptoms | 16.6 (15.1-18.0) | 35.2 (30.4-40.1) | 46.5 (42.3-50.6) | 69.0 (63.2-74.8) |
| PTSD | 11.7 (10.2-13.3) | 27.9 (22.2-33.7) | 56.1 (52.2-60.1) | 65.9 (58.8-72.9) |
| Fear of recurrence | 17.8 (16.0-19.6) | 28.4 (23.7-33.1) | 53.5 (48.2-58.9) | 57.1 (48.4-65.8) |
| Raw scores (range) |  |  |  |  |
| Physical fatigue (0-21) | 7.1 (6.7-7.4) | 13.3 (12.3-14.3) | 9.6 (8.8-10.4) | 16.2 (14.8-17.5) |
| Mental fatigue (0-12) | 3.7 (3.6-3.9) | 6.2 (5.7-6.7) | 4.9 (4.4-5.4) | 8.4 (7.6-9.1) |
| Depressive symptoms (0-15) | 1.3 (1.1-1.5) | 5.4 (4.5-6.2) | 4.7 (4.1-5.3) | 10.8 (10.0-11.7) |
| Anxiety symptoms (0-20) | 3.3 (3.0-3.6) | 7.0 (6.1-8.0) | 9.3 (8.5-10.1) | 13.8 (12.6-15.0) |
| PTSD (0-24) | 2.8 (2.4-3.2) | 6.7 (5.3-8.1) | 13.5 (12.5-14.4) | 15.8 (14.1-17.5) |
| Fear of recurrence (0-15) | 2.7 (2.4-2.9) | 4.3 (3.6-5.0) | 8.0 (7.2-8.8) | 8.6 (7.3-9.9) |

^a^ Proportions are latent profile analysis estimated proportions.
^b^ All scores converted to 0-100 scales for comparability.
Questionnaires: Fatigue: Chalder’s Fatigue Score; Depressive symptoms: Patient Health Questionnaire-9; Anxiety symptoms: The Hospital Anxiety and Depression Scale-9, Anxiety subscale; PTSS: Impact of Event Scale-6; Fear of (cancer) recurrence: Assessment of Survivor Concerns Scale.

Abbreviations: CI: confidence interval.

Supplemental table 8: (Childhood cancer survivors only) Fit statistics and classification coefficients for all latent profile models

| K | LL | AIC | BIC | BF | VLMR-LRT | Entropy | PPmax | est.%min |
| --- | --- | --- | --- | --- | --- | --- | --- | --- |
| 1 | -9452 | 18927 | 18980 | <0.001 | - | - | 1.00 (0.00) | 100.0% |
| 2 | -8865 | 17767 | 17851 | <0.001 | <0.001 | **0.911** | 0.97 (0.08) | 26.9% |
| 3 | -8728 | 17509 | 17623 | <0.001 | <0.001 | 0.915 | 0.96 (0.09) | 5.1% |
| 4 | -8649 | 17364 | 17509 | <0.001 | <0.001 | 0.890 | 0.94 (0.12) | 4.8% |
| 5 | -8585 | 17250 | 17426 | **66.048** | <0.001 | 0.844 | 0.90 (0.14) | 3.9% |
| 6 | -8567 | 17228 | 17435 | <0.001 | <0.001 | 0.871 | 0.91 (0.14) | 3.7% |
| 7 | **-8532** | **17171** | **17408** |  | <0.001 | 0.820 | 0.86 (0.15) | 4.0% |

Note: Bold values indicate “best” fit.

Abbreviations: AIC = Aikaike information criterion; BF = Bayes factor; BIC = Bayesian information criterion; est.%_min_ = model estimated proportion of participants in the smallest class; K = number of classes; LL = log-likelihood; PP_max_ = mean propensity probability of the single profile with highest probability (where participants were allocated by modal assignment); VLMR-LRT = Vuong-Lo-Mendell-Rubin adjusted likelihood-ratio-test.

Supplemental table 9: (Childhood cancer survivors only) Background characteristics of participants allocated to the four identified latent profiles (N=598)

|  | Low | Moderate fatigue/  high anxiety | High fatigue/  moderate distress | High | p-value^a^ |
| --- | --- | --- | --- | --- | --- |
|  | N=392  (65.6%) | N=86  (14.4%) | N=92  (15.4%) | N=28  (4.7%) |  |
| Background |  |  |  |  | 0.023 |
| Age at study (yrs) | 30.1 (±8.0) | 30.8 (±7.9) | 31.1 (±7.4) | 27.0 (±6.9) | 0.090 |
| Sex, female | 201 (51%) | 51 (59%) | 65 (71%) | 20 (71%) | 0.002 |
| Highest achieved education |  |  |  |  | <0.001 |
| Compulsory school | 16 (4%) | 4 (5%) | 4 (4%) | 7 (25%) |  |
| Vocational school | 87 (22%) | 12 (14%) | 27 (29%) | 8 (29%) |  |
| High school | 64 (16%) | 21 (25%) | 13 (14%) | 4 (14%) |  |
| University | 224 (57%) | 48 (56%) | 48 (52%) | 9 (32%) |  |
| Cancer related |  |  |  |  | 0.52 |
| Age at diagnosis (yrs) | 10.5 (±6.0) | 10.7 (±6.2) | 11.2 (±5.5) | 8.4 (±6.2) | 0.20 |
| Time since first diagnosis | 19.0 (±6.7) | 19.5 (±6.9) | 19.3 (±6.3) | 18.0 (±5.8) | 0.74 |
| Primary diagnosis group |  |  |  |  | 0.013 |
| Leukemias | 115 (29%) | 24 (28%) | 36 (39%) | 16 (57%) |  |
| Lymphomas | 98 (25%) | 23 (27%) | 17 (18%) | 6 (21%) |  |
| Bone and soft tissue sarcomas | 44 (11%) | 18 (21%) | 10 (11%) | 2 (7%) |  |
| Germ cell tumors | 50 (13%) | 4 (5%) | 13 (14%) | 0 (0%) |  |
| Other | 85 (22%) | 17 (20%) | 16 (17%) | 4 (14%) |  |
| Treatment^b^ |  |  |  |  | 0.012 |
| Local treatment | 52 (13%) | 11 (13%) | 6 (7%) | 0 (0%) |  |
| Systemic single treatment | 103 (27%) | 20 (24%) | 24 (26%) | 15 (56%) |  |
| Multiple treatments | 231 (60%) | 52 (63%) | 61 (67%) | 12 (44%) |  |
| Recurring or second cancer(s) | 3 (1%) | 2 (2%) | 2 (2%) | 1 (4%) | 0.37 |

^a^ p-values calculated from univariable linear regression models for continuous variables and chi-squared statistics for categorical variables.
^b^ Treatment was hierarchically aggregated into minimal treatment for melanoma (local treatment only and diagnosis of melanoma), local treatment (local radiotherapy or surgery only), systemic single treatment (including chemotherapy, stem cell transplantation and immune therapy) or multiple treatments.
Data are presented as mean (±SD) for continuous variables, and n (%) for categorical variables.

Abbreviations: SD: standard deviation; yrs: years.

## Sensitivity analysis 3: Latent profile analysis and related characteristics in young adult cancer survivors only

Supplemental figure 5: (Young adult survivors only) Estimated marginal mean T-scores for fatigue and psychological symptom burdens for the four identified latent profiles (N=1295)


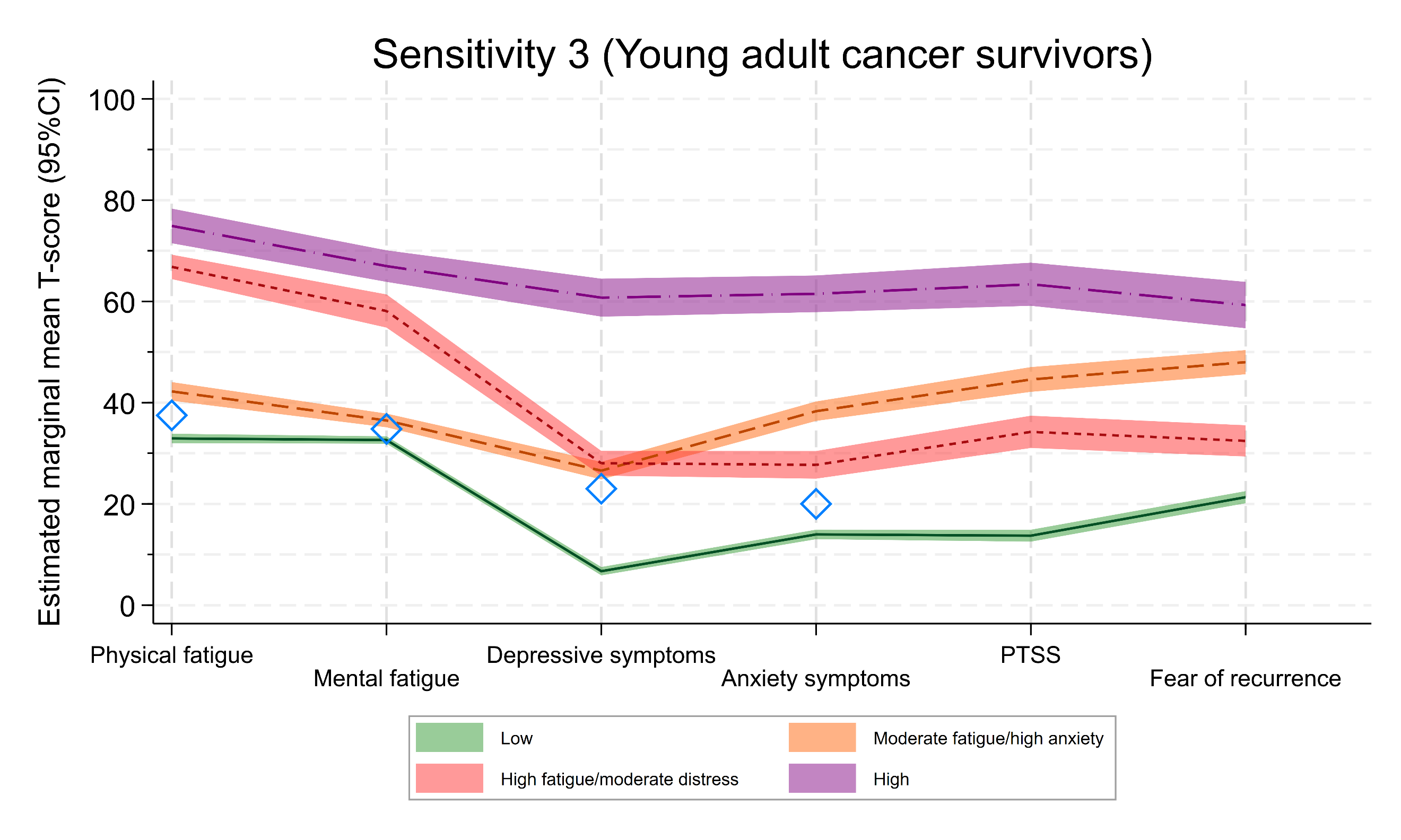


The figure shows results from the latent profile analysis where young adult cancer survivors were included with marginal mean T-scores (darker solid and dashed lines) with 95% confidence intervals (light colored areas) for each of the four identified profiles. T-score is a standardized scale from 0 to 100 for all outcomes.

**◇** Normative scores for fatigue, depressive symptoms, and anxiety symptoms from Norwegian populations ^1,2^

Abbreviations: PTSS: post-traumatic stress symptoms.

Supplemental table 10: (Young adult survivors only) Estimated marginal means (95%CI) for fatigue and psychological symptom burdens for the four identified latent profiles

| Estimated proportion^a^ | Low  (62.0%) | Moderate fatigue/ high anxiety (11.4%) | High fatigue/ moderate distress (21.8%) | High  (4.9%) |
| --- | --- | --- | --- | --- |
|  | Mean (95% CI) | Mean (95% CI) | Mean (95% CI) | Mean (95% CI) |
| Standardized 0-100 T-scores^b^ |  |  |  |  |
| Physical fatigue | 32.9 (32.0-33.9) | 66.8 (64.4-69.2) | 42.2 (40.4-44.1) | 74.9 (71.5-78.3) |
| Mental fatigue | 32.6 (31.9-33.4) | 58.1 (54.8-61.4) | 36.5 (35.1-37.8) | 67.0 (63.9-70.1) |
| Depressive symptoms | 6.7 (5.9-7.5) | 28.0 (25.6-30.5) | 26.6 (24.8-28.3) | 60.7 (57.0-64.5) |
| Anxiety symptoms | 14.0 (13.0-14.9) | 27.7 (25.0-30.4) | 38.3 (36.4-40.2) | 61.5 (57.9-65.1) |
| PTSD | 13.7 (12.5-14.9) | 34.2 (31.0-37.4) | 44.6 (42.1-47.0) | 63.4 (59.1-67.6) |
| Fear of recurrence | 21.3 (20.1-22.5) | 32.5 (29.4-35.5) | 48.0 (45.6-50.4) | 59.3 (54.7-63.9) |
| Raw scores (range) |  |  |  |  |
| Physical fatigue (0-21) | 6.9 (6.7-7.1) | 14.0 (13.5-14.5) | 8.9 (8.5-9.3) | 15.7 (15.0-16.4) |
| Mental fatigue (0-12) | 3.9 (3.8-4.0) | 7.0 (6.6-7.4) | 4.4 (4.2-4.5) | 8.0 (7.7-8.4) |
| Depressive symptoms (0-15) | 1.0 (0.9-1.1) | 4.2 (3.8-4.6) | 4.0 (3.7-4.2) | 9.1 (8.5-9.7) |
| Anxiety symptoms (0-20) | 2.8 (2.6-3.0) | 5.5 (5.0-6.1) | 7.7 (7.3-8.0) | 12.3 (11.6-13.0) |
| PTSD (0-24) | 3.3 (3.0-3.6) | 8.2 (7.4-9.0) | 10.7 (10.1-11.3) | 15.2 (14.2-16.2) |
| Fear of recurrence (0-15) | 3.2 (3.0-3.4) | 4.9 (4.4-5.3) | 7.2 (6.8-7.6) | 8.9 (8.2-9.6) |

^a^ Proportions are latent profile analysis estimated proportions.
^b^ All scores converted to 0-100 scales for comparability.
Questionnaires: Fatigue: Chalder’s Fatigue Score; Depressive symptoms: Patient Health Questionnaire-9; Anxiety symptoms: The Hospital Anxiety and Depression Scale-9, Anxiety subscale; PTSS: Impact of Event Scale-6; Fear of (cancer) recurrence: Assessment of Survivor Concerns Scale.

Abbreviations: CI: confidence interval.

Supplemental table 11: (Young adult survivors only) Fit statistics and classification coefficients for all latent profile models

| K | LL | AIC | BIC | BF | VLMR-LRT | Entropy | PPmax | est.%min |
| --- | --- | --- | --- | --- | --- | --- | --- | --- |
| 1 | -19638 | 39299 | 39361 | 0.000 | - | - | 1.00 (0.00) | 100.0% |
| 2 | -18495 | 37028 | 37126 | <0.001 | 0 | 0.883 | 0.97 (0.09) | 26.4% |
| 3 | -18141 | 36334 | 36469 | <0.001 | <0.001 | 0.871 | 0.94 (0.11) | 7.3% |
| 4 | -17946 | 35959 | 36129 | <0.001 | <0.001 | **0.889** | 0.94 (0.12) | 4.9% |
| 5 | -17854 | 35788 | 35995 | <0.001 | <0.001 | 0.860 | 0.91 (0.14) | 3.8% |
| 6 | -17769 | 35631 | 35874 | <0.001 | <0.001 | 0.876 | 0.91 (0.13) | 3.4% |
| 7 | **-17685** | **35478** | **35757** |  | <0.001 | 0.877 | 0.90 (0.14) | 3.3% |

Note: Bold values indicate “best” fit.

Abbreviations: AIC = Aikaike information criterion; BF = Bayes factor; BIC = Bayesian information criterion; est.%_min_ = model estimated proportion of participants in the smallest class; K = number of classes; LL = log-likelihood; PP_max_ = mean propensity probability of the single profile with highest probability (where participants were allocated by modal assignment); VLMR-LRT = Vuong-Lo-Mendell-Rubin adjusted likelihood-ratio-test.

Supplemental table 12: (Young adult survivors only) Background characteristics of participants allocated to the four identified latent profiles (N=1295)

|  | Low | Moderate fatigue/  high anxiety | High fatigue/  moderate distress | High | p-value^a^ |
| --- | --- | --- | --- | --- | --- |
|  | N=813  (62.8%) | N=139  (10.7%) | N=282  (21.8%) | N=61  (4.7%) |  |
| Background |  |  |  |  |  |
| Age at study (yrs) | 49.7 (±7.9) | 47.9 (±7.6) | 48.1 (±7.7) | 46.4 (±7.5) | <0.001 |
| Sex, female | 565 (69%) | 109 (78%) | 218 (77%) | 51 (84%) | 0.004 |
| Highest achieved education |  |  |  |  | 0.049 |
| Compulsory school | 39 (5%) | 13 (9%) | 23 (8%) | 5 (8%) |  |
| Vocational school | 189 (23%) | 36 (26%) | 85 (30%) | 14 (23%) |  |
| High school | 75 (9%) | 12 (9%) | 30 (11%) | 5 (8%) |  |
| University | 505 (63%) | 77 (56%) | 142 (51%) | 37 (61%) |  |
| Cancer related |  |  |  |  |  |
| Age at diagnosis (yrs) | 32.8 (±5.3) | 32.9 (±5.6) | 32.8 (±5.3) | 33.0 (±5.4) | 0.870 |
| Time since first diagnosis (yrs) | 16.3 (±6.8) | 14.4 (±6.3) | 14.7 (±6.6) | 12.8 (±6.4) | <0.001 |
| Primary diagnosis group |  |  |  |  | 0.001 |
| Melanoma | 219 (27%) | 19 (14%) | 53 (19%) | 7 (11%) |  |
| Breast cancer | 278 (34%) | 60 (43%) | 118 (42%) | 32 (52%) |  |
| Colorectal cancer | 92 (11%) | 18 (13%) | 26 (9%) | 7 (11%) |  |
| Non-Hodgkin's lymphoma | 138 (17%) | 33 (24%) | 50 (18%) | 9 (15%) |  |
| Acute lymphatic leukemia | 86 (11%) | 9 (6%) | 35 (12%) | 6 (10%) |  |
| Treatment^b^ |  |  |  |  | <0.001 |
| Minimal treatment (melanoma) | 194 (25%) | 15 (12%) | 46 (17%) | 7 (12%) |  |
| Local treatment | 90 (12%) | 9 (7%) | 24 (9%) | 4 (7%) |  |
| Systemic single treatment | 84 (11%) | 9 (7%) | 33 (13%) | 6 (11%) |  |
| Multiple treatments | 394 (52%) | 94 (74%) | 160 (61%) | 40 (70%) |  |
| Recurring or second cancer(s) | 35 (4%) | 8 (6%) | 9 (3%) | 2 (3%) | 0.640 |

^a^ p-values calculated from univariable linear regression models for continuous variables and chi-squared statistics for categorical variables.
^b^ Treatment was hierarchically aggregated into minimal treatment for melanoma (local treatment only and diagnosis of melanoma), local treatment (local radiotherapy or surgery only), systemic single treatment (including chemotherapy, stem cell transplantation and immune therapy) or multiple treatments.
Data are presented as mean (±SD) for continuous variables, and n (%) for categorical variables.

Abbreviations: SD: standard deviation; yrs: years.

# References

1. Dahl AA, Grotmol KS, Hjermstad MJ, Kiserud CE, Loge JH. Norwegian reference data on the Fatigue Questionnaire and the Patient Health Questionnaire-9 and their interrelationship. *Ann Gen Psychiatry*. 2020;19(1):60. doi:10.1186/s12991-020-00311-5

2. Grov EK, Dahl AA, Moum T, Fosså SD. Anxiety, depression, and quality of life in caregivers of patients with cancer in late palliative phase. *Ann Oncol*. 2005;16(7):1185-1191. doi:10.1093/annonc/mdi210

3. Nylund KL, Asparouhov T, Muthén BO. Deciding on the Number of Classes in Latent Class Analysis and Growth Mixture Modeling: A Monte Carlo Simulation Study. *Struct Equ Modeling*. 2007;14(4):535-569. doi:10.1080/10705510701575396

4. Weller BE, Bowen NK, Faubert SJ. Latent Class Analysis: A Guide to Best Practice. *Journal of Black Psychology*. 2020;46(4):287-311. doi:10.1177/0095798420930932

5. van de Schoot R, Sijbrandij M, Winter SD, Depaoli S, Vermunt JK. The GRoLTS-Checklist: Guidelines for Reporting on Latent Trajectory Studies. *Struct Equ Modeling*. 2017;24(3):451-467. doi:10.1080/10705511.2016.1247646

6. Asparouhov T, Muthen B. Variable-Speciﬁc Entropy Contribution.

7. Lo Y, Mendell NR, Rubin DB. Testing the number of components in a normal mixture. *Biometrika*. 2001;88(3):767-778. doi:10.1093/biomet/88.3.767

8. Masyn KE. Latent class analysis and finite mixture modeling. In: *The Oxford Handbook of Quantitative Methods: Statistical Analysis, Vol. 2*. Oxford library of psychology. Oxford University Press; 2013:551-611.

9. Nylund-Gibson K. Ten frequently asked questions about latent class analysis. *Translational Issues in Psychological Science*. 20181213;4(4):440. doi:10.1037/tps0000176
